# ^99m^TcO_4_^−^-, Auger-Mediated Thyroid Stunning: Dosimetric Requirements and Associated Molecular Events

**DOI:** 10.1371/journal.pone.0092729

**Published:** 2014-03-24

**Authors:** Béatrice Cambien, Philippe R. Franken, Audrey Lamit, Thibault Mauxion, Peggy Richard-Fiardo, Julien Guglielmi, Lydie Crescence, Bernard Mari, Thierry Pourcher, Jacques Darcourt, Manuel Bardiès, Georges Vassaux

**Affiliations:** 1 Laboratoire TIRO, UMRE 4320, iBEB, DSV, CEA, Nice, France; 2 Université de Nice-Sophia Antipolis, Nice, France; 3 Centre Antoine Lacassagne, Department of nuclear medicine, Nice, France; 4 UMR 1037 INSERM/UPS, Centre de Recherche en Cancérologie, Toulouse, France; 5 Institut de Pharmacologie Moléculaire et Cellulaire-IPMC, CNRS UMR 7275, Sophia Antipolis, France; RIKEN Advanced Science Institute, JAPAN

## Abstract

Low-energy Auger and conversion electrons deposit their energy in a very small volume (a few nm^3^) around the site of emission. From a radiotoxicological point of view the effects of low-energy electrons on normal tissues are largely unknown, understudied, and generally assumed to be negligible. In this context, the discovery that the low-energy electron emitter, ^99m^Tc, can induce stunning on primary thyrocytes *in vitro*, at low absorbed doses, is intriguing. Extrapolated *in vivo*, this observation suggests that a radioisotope as commonly used in nuclear medicine as ^99m^Tc may significantly influence thyroid physiology. The aims of this study were to determine whether ^99m^Tc pertechnetate (^99m^TcO_4_^−^) is capable of inducing thyroid stunning *in vivo*, to evaluate the absorbed dose of ^99m^TcO_4_^−^ required to induce this stunning, and to analyze the biological events associated/concomitant with this effect. Our results show that ^99m^TcO_4_^−^–mediated thyroid stunning can be observed *in vivo* in mouse thyroid. The threshold of the absorbed dose in the thyroid required to obtain a significant stunning effect is in the range of 20 Gy. This effect is associated with a reduced level of functional Na/I symporter (NIS) protein, with no significant cell death. It is reversible within a few days. At the cellular and molecular levels, a decrease in NIS mRNA, the generation of double-strand DNA breaks, and the activation of the p53 pathway are observed. Low-energy electrons emitted by ^99m^Tc can, therefore, induce thyroid stunning *in vivo* in mice, if it is exposed to an absorbed dose of at least 20 Gy, a level unlikely to be encountered in clinical practice. Nevertheless this report presents an unexpected effect of low-energy electrons on a normal tissue *in vivo*, and provides a unique experimental setup to understand the fine molecular mechanisms involved in their biological effects.

## Introduction

Thyroid stunning is a clinical problem in which exposure of a patient to diagnostic amounts of ^131^I has been described to alter the ability of differentiated thyroid carcinoma, or remnants of thyroid tissue after thyroidectomy, to take up therapeutic, irradiating doses of ^131^I [1], [2], [3], [4], [5]. Although widely documented in the literature, the reality and existence of this phenomenon are questioned by many clinicians [5]. Some experts advocate that the so-called stunning effect is in fact the consequence of an early destruction of the target tissues by an excessive amount of radiotracer used for a diagnostic purpose [5]. In this clinical context, basic scientific studies using cultured, polarized thyroid epithelial cells *in vitro* have recently produced clear and convincing evidence of the existence of a stunning effect and of its mechanism [6], [7], [8], [9], [10]. These studies demonstrated that irradiation of the cell culture via ^131^I uptake resulted in a significant reduction in basal-to-apical iodide transport, even at low absorbed doses. This effect reached a 50% reduction in iodide transport at an absorbed dose of around 1.5 Gy and was observed with no evidence of cell death [7]. Using the same experimental setup, the same group demonstrated that ^131^I-induced stunning could be explained by a decrease in the mRNA level of the Na/I symporter (NIS), the transmembrane protein involved in the iodide uptake by the thyroid [8]. More recently, the same group published an intriguing study in which the “stunning efficacies” of ^131^I, ^123^I, ^99m^Tc and ^211^As were compared in the same cell culture system [9]. Experiments were designed to deliver a “standardized” absorbed dose of 0.5 Gy from these radioisotopes, which are all accumulated in thyroid cells via a NIS-dependent mechanism. The results showed that all of these radioisotopes were capable of reducing iodide transport by decreasing NIS mRNA levels a few days later. The “stunning power” of ^131^I appeared to be poorer than that of ^123^I or even ^99m^Tc in this setting. Although the absorbed dose-rate resulting from the relative half-life and radiation type of these different isotopes may be partially responsible for the difference in biological activity observed, these results are puzzling. Extrapolated *in vivo*, this study suggests that a radioisotope as commonly used in nuclear medicine as ^99m^Tc may significantly influence thyroid biology. In addition, SPECT/CT imaging of ectopic NIS gene expression with ^99m^TcO_4_^−^ has been validated at the preclinical level and is starting to be used in the clinic to determine the kinetics of gene transfer in gene therapy [11], [12], [13], [14]. Hence, the conditions required to induce a ^99m^TcO_4_^−^-mediated thyroid stunning effect *in vivo* need to be clarified. The stunning effect of ^99m^Tc is attributed to low-energy electrons.

The energy of low-energy electrons is deposited in a very small volume (a few nm^3^) around the site of de-excitation of radionuclides such as ^123^I, ^125^I, ^111^In, or ^99m^Tc [15], [16]. In biology, these electrons are thought to have played a significant role as mutagenic agents in evolution [17] and today they represent the basis of attractive strategies for new, targeted cancer treatments [16]. In the latter approaches, Auger electron emitters (typically ^125^I or ^111^In) are incorporated into small molecules [18], peptides [19], proteins [20], [21] or antibodies [22] capable of targeting cancer cell nuclei, where Auger electrons will exert their irradiating effects. From a radiotoxicological point of view, however, the effects of Auger electrons on normal, nonpathological biological systems are largely unknown, understudied and generally assumed to be negligible [23].

The aims of the present study were to determine whether ^99m^TcO_4_^−^ was capable of inducing stunning *in vivo*, to evaluate the absorbed dose of ^99m^TcO_4_^−^ required to induce this stunning, and to analyze the biological events associated/concomitant with this effect.

## Materials and Methods

### Animals

Female, eight-week-old Balb/c mice were obtained from Janvier (Le Genest Saint Isle, France). Animal housing and procedures were conducted according to the French Agriculture Ministry guidelines and were approved by the local ethics committee (CIEPAL: Comité Institutionnel d'Ethique Pour l'Animal de Laboratoire) (Permit number # A06-088-14-138). Animals were fed using a standard regimen with a normal-iodide diet, except for two animals included in the protocol for dosimetric calculations that received an iodide-poor regimen for 8 days before the study (see below).

### *In vivo* MicroSPECT/CT Studies

**Figure 1:**
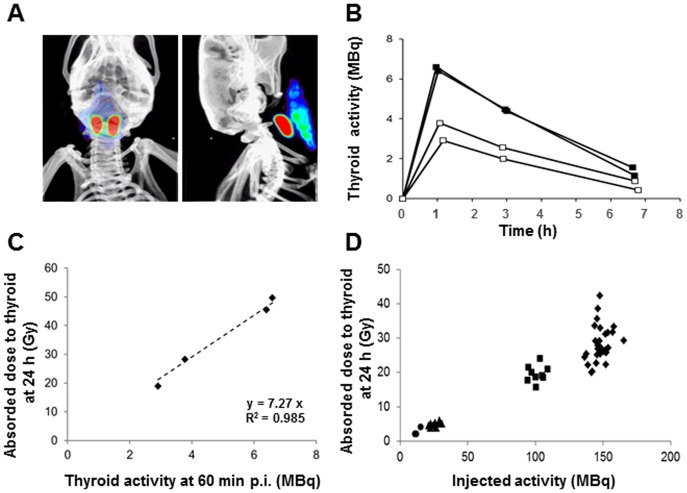
^99m^TcO_4_^−^-SPECT/CT imaging and absorbed dose to the thyroid. (A) Volume-rendered, fused SPECT/CT images in anterior and lateral views demonstrating the thyroid lobes (red) and the salivary glands (blue-green), 60 minutes after ^99m^TcO_4_^−^ administration. (B) Kinetics of ^99m^TcO_4_^−^ activity in the thyroids of four Balb/c mice fed with a normal iodide diet (open squares) or with a low iodide diet (filled squares), obtained upon injection of 150 MBq of ^99m^TcO_4_^−^. The values presented are not corrected for decay. (C) Correspondence between thyroid activity (MBq) at 60 minutes post injection and absorbed dose to the thyroid (Gy) at 24 hours. (D) Absorbed dose to the thyroid at 24 hours in mice injected with 10 MBq (circles, n = 4), 25 MBq (triangles, n = 9), 100 MBq (squares, n = 9) or 150 MBq (diamonds, n = 29) ^99m^TcO_4_^−^.

^99m^Tc pertechnetate (^99m^TcO_4_^−^) was obtained from a freshly eluted ^99^Mo/^99m^Tc generator. Animals were administered intraperitoneally with ^99m^TcO_4_^−^ activities ranging from 10 MBq to 150 MBq. Thyroid tracer uptake was measured 60 minutes later using a dedicated microSPECT/CT scanner (eXplore speCZT CT120, GE) under gas anesthesia (air and 1-2% isoflurane) in an air-warmed imaging chamber (Minerve, Esternay, France) to keep body temperature constant at 37°C. The SPECT scanner uses a stationary full ring of CZT detectors and a rotating 7-pinhole (1 mm opening) collimator. A total of 350 projections were acquired over 360° in 8 minutes. Images were reconstructed using the manufacturer's 3D-OSEM algorithm (5 subsets and 11 iterations), which incorporates the system's collimator–detector response function and scatter correction. SPECT imaging system calibration for ^99m^Tc volume sensitivity was obtained by correlation of the results with a calibrated well counter using the NEMA protocols [24]. Reconstructed images were analyzed and quantified using AMIDE software [25]. Tracer activity in the thyroid was calculated by converting the total image units measured in a 3D region of interest to MBq using the calibration factor. Thyroid uptake was expressed as percentage of the injected dose (%ID) after decay correction, except for dosimetric calculations (see Figure 1B).

### Animal Study Protocols

For dosimetric calculations, thyroid SPECT imaging was repeated 1, 3 and 7 hours after intraperitoneal administration of 150 MBq ^99m^TcO_4_^−^ in four mice. Two mice had a standard-regimen diet, two had a poor-iodide-regimen diet for 8 days before the study. This dietary manipulation is known to lead to different thyroid ^99m^TcO_4_^−^ uptake capacity and was used to establish a correspondence between absorbed dose to the thyroid (at 24 hours) and thyroid activity at the peak.

To determine the early effect of an initial administration of ^99m^TcO_4_^−^ on thyroid uptake, 21 animals that received 25 MBq (n = 3), 100 MBq (n = 3) or 150 MBq (n = 15) were imaged again 24 hours after the initial SPECT, using a standard (50 MBq) ^99m^TcO_4_^−^ activity.

To investigate the later impact of the administration of ^99m^TcO_4_^−^ on the thyroid uptake, 12 animals initially received 150 MBq tracer, resulting in an absorbed dose to the thyroid of 27.5±4.1 Gy, ranging from 20.4 to 35.7 Gy. The animals were imaged again 1 (n = 3), 2 (n = 3), 4 (n = 3) or 8 days (n = 3) later, using a standard ^99m^TcO_4_^−^ activity (50 MBq). After the end of the second scan, the animals were culled and, when relevant, biopsies of different organs were collected.

### Dosimetry

The absorbed dose in the thyroid (in Gy) was estimated based on the MIRD formalism [26]. The cumulated activity in the thyroid (Bq.s) was calculated based on quantitative imaging and multiplied by the thyroid self S-value (Gy.Bq^−1^.s^−1^) obtained from Monte Carlo simulation of a representative mouse model. The kinetics of ^99m^TcO_4_^−^ activity in the thyroid are presented in Figure 1B. Activities were not corrected for nuclear decay. Cumulated activity was calculated for each mouse, using a linear fitting model for the uptake phase, and a mono-exponential fitting model for the washout, both implemented in Root software (http://root.cern.ch). No activity was considered at t = 0 and the endpoint for cumulated activity calculation was taken at t = 24 hours.

S-values were calculated based on the realistic-digital-mouse (Moby) whole-body phantoms (version 2), representing a 16-week-old male C57BL/6 mouse [27]. We generated a 22 g mouse model as a 3D rectangular matrix of cubic voxels (200×200×200 μm^3^). The final 3D image dataset was composed of 256×550×256 voxels and saved in a raw format (16-bit; unsigned integer; little-endian; 72 MB). Thyroid mass was reduced to 5.4 mg by applying an erode mask implemented on ImageJ software. Soft tissues, lungs, bones and air density, and material composition [28] were also taken into account for radiation transport purposes.

S-values were calculated using Monte Carlo modeling of radiation transport and energy deposition in the voxel-based mouse model. Up to 10^6^ particles were simulated using GATE (version 6.1), based on the GEANT4 toolkit (version 9.04 patch01), both of which are well-established codes for radiation transport [29], [30], [31].

The voxel-based mouse was implemented with the *CompressedMatrix* option, the most suited function available for dosimetric purposes in that version, and regions of interest were defined using the *range* option. *Physics List Standard Option 3* was used to define physics processes. The deposited energy was scored at the voxel level of the phantom with the DoseActor, *doseDistributionEdep*. Statistical uncertainties were calculated using the associated *UncertaintyEdep* option. GATE was run with Mersenne Twister [32].

The thyroid self S-value was calculated for ^99m^Tc. All detailed photon and electron emissions were based on the “MIRD radionuclide data and decay schemes” [33]. Cubic spline interpolation was applied to all continuous energy spectra used in simulations. The source was assumed to be distributed homogeneously within the thyroid and statistical uncertainties in self S-values were kept below 1%.

### Membrane Vesicle Preparation And SDS-PAGE Analyses

Thyroid membrane proteins were obtained as previously described [34] and subjected to SDS-PAGE electrophoresis. Western blotting was performed with antibody 25 anti-mouse NIS, an affinity-purified rabbit immunoreactive serum fraction, as previously described [35], an anti-Ser139 phospho H2AX mouse monoclonal Ab (Millipore, France), or with an anti-β-actin antibody (Sigma).

### RNA Extraction, Quantitative RT-PCR and Microarrays

Extraction of total RNA from mouse thyroid, and quantitative RT-PCR, were performed as previously described [36], [37], [38]. Relative mRNA expression levels were determined using ΔCt values obtained by subtracting Ct control (mouse actin) from Ct target gene (mouse NIS), and expressed using the comparative CT method (2^−ΔCT^).

For microarray analysis, RNA samples were labeled with Cy3 dye using the Low RNA Input QuickAmp Kit (Agilent), as recommended by the supplier. Labeled cRNA probes (400 ng) were hybridized on 8×60K high-density SurePrint G3 gene mouse GE 8×60K Agilent microarrays. Four biological replicates were performed for each experimental condition. The experimental data are deposited in the NCBI Gene Expression Omnibus (GEO) (http://www.ncbi.nlm.nih.gov/geo/) under the record number, GSE46470. Normalization of microarray data was performed using the Limma package (http://www.bioconductor.org). Interslide normalization was performed using the quantile methods. Means of ratios from ^99m^Tc-treated versus control tissues were calculated and B-test analysis was performed. Differentially expressed genes were selected based on an adjusted p value of 0.05. Data from expression microarrays were analyzed for enrichment in biological themes (Gene Ontology molecular function and canonical pathways) and biological networks were built using Ingenuity Pathway Analysis software (http://www.ingenuity.com/) and Mediante [39].

### Histology/Immunohistochemistry

Formalin-fixed, paraffin-embedded thyroid and salivary gland tissue sections were stained with hematoxylin/eosin for morphologic evaluation. Mouse NIS and phospho-Histone H2AX immunostaining were performed using a rabbit polyclonal anti-NIS antibody (antibody 25, see [35]) and an anti-Ser139 phospho H2AX rabbit mAb (Cell Signalling, France), followed by HRP-conjugated anti-rabbit antibodies and the avidin-biotin complex immunoperoxidase method, respectively. Apoptosis (TUNEL) staining was performed using the pro-TUNEL assay kit (Euromedex, France). The sections were analyzed by a certified veterinary pathologist.

### Statistical Analysis

Dual comparisons were made using the Student's t-test and comparisons between multiple conditions were analyzed using ANOVA. Statistical significance was set at p<0.05.

## Results

### Dosimetric calculations

Figure 1A presents typical, volume-rendered, fused SPECT/CT images in anterior and lateral views of the thyroid region. ^99m^TcO_4_^−^ is accumulated in the thyroid (red) and the salivary gland (blue-green). To measure precisely the thyroid ^99m^TcO_4_^−^ content, regions-of-interest excluding the salivary glands were drawn around the entire thyroid. The kinetics of ^99m^TcO_4_^−^ uptake and release in the thyroid were measured by SPECT/CT on live, anesthetized mice, 60 minutes, three hours, and seven hours after intraperitoneal administration of 150 MBq ^99m^TcO_4_^−^. Figure 1B shows that the radioisotope content in the thyroid reaches its maximal value within one hour after ^99m^TcO_4_^−^ injection, thereafter decreasing progressively to reach near-basal levels at an estimated time of 8 hours. To enrich the dataset, we performed a similar experiment on animals that were fed on a poor-iodide diet. Accumulation of ^99m^TcO_4_^−^ in the thyroid followed the same overall pattern (Figure 1B) but the value at the peak averaged 6.5 MBq with a low-iodide diet while it averaged 3.5 MBq in mice fed a normal-iodide diet. Dosimetric calculations based on these pharmacokinetic data are detailed in Material S1. They indicate that the thyroid self S-value was 4.55E^−10^ Gy.Bq^−1^.s^−1^. In these experimental conditions, the peak activity measured in the thyroid (ranging from 3 to 7 MBq) was strongly correlated (R^2^ = 0.985) with the cumulated activity, and hence with the absorbed dose to the thyroid at 24 hours (range 18.9 to 49.6 Gy) (Figure 1C). Based on these kinetics and calculations, SPECT/CT analyses of thyroid activity throughout the study were systematically performed 60 minutes after radioisotope administration and, when relevant, transformed into absorbed doses. Absorbed doses to the thyroid at 24 hours averaged 2.6±1.0, 4.8±0.7, 19.6±2.4, and 28.4±5.2 Gy, for injected activities of 10, 25, 100 and 150 MBq, respectively (Figure 1D). Thyroid activity (expressed as %ID) was 2.84±0.63, 2.6±0.35, 2.67±0.33 and 2.63±0.47 in mice injected with 10 (n = 4), 25 (n = 9), 100 (n = 9) or 150 (n = 29) MBq ^99m^TcO_4_^−^, respectively. MBq, respectively (Figure 1D).

### Dosimetric Requirement For ^99m^TcO_4_^−^-Mediated Thyroid Stunning

**Figure 2:**
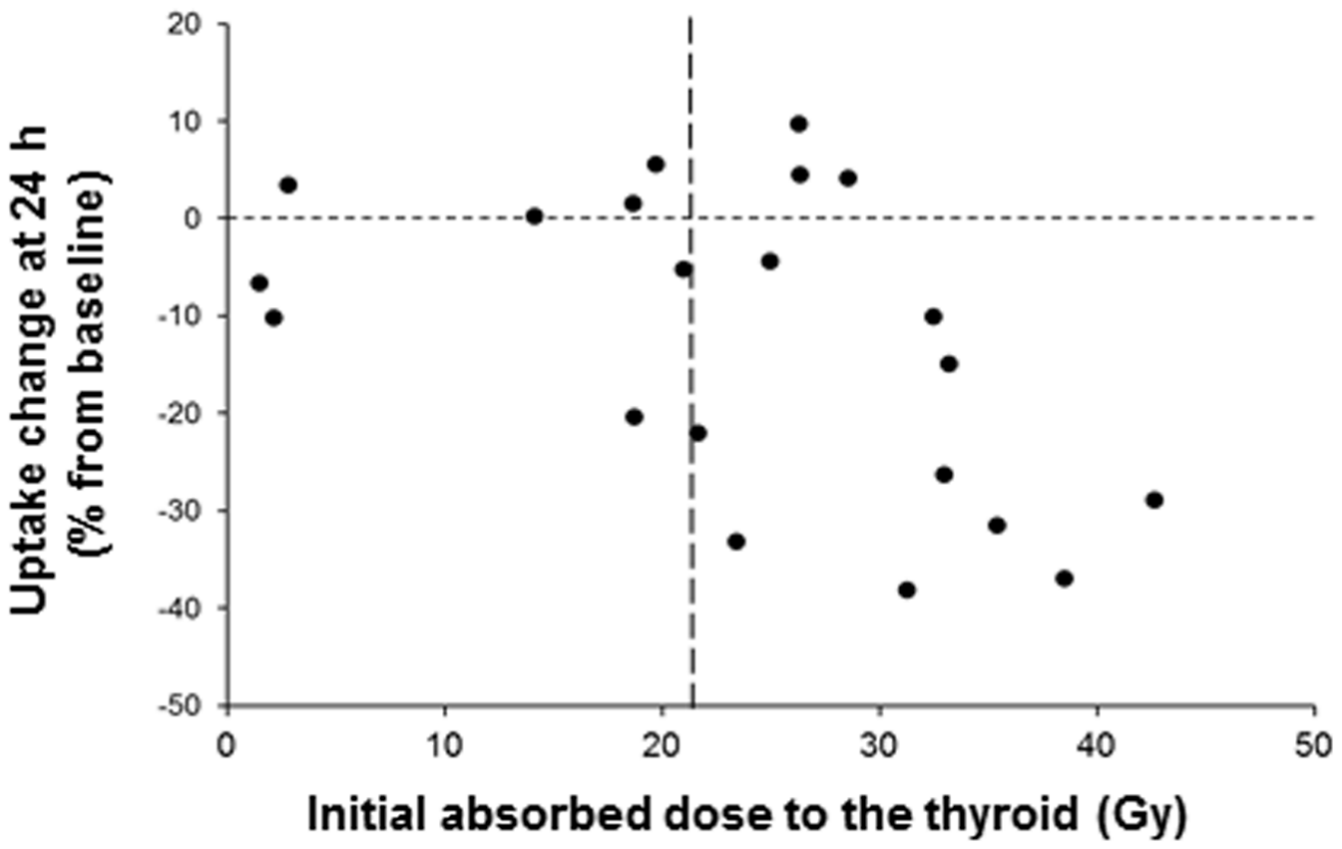
Changes in thyroid uptake 24^99m^TcO_4_^−^. Mice were injected with various amounts of ^99m^TcO_4_^−^ (25 to 150 MBq). Twenty-four hours later, a standard dose of 50 MBq ^99m^TcO_4_^−^ was injected to determine the capacity of the thyroid to take up the radioisotope. The figure represents the relationship between the initial absorbed dose to the thyroid (in Gy) and the percent change in ^99m^TcO_4_^−^ uptake 24 hours later.

Figure 2 shows that the threshold of initial absorbed dose required to observe a significant reduction (p = 0.048) in the capacity of the thyroid to take up ^99m^TcO_4_^−^ one day later was 22 Gy. In animals that received less than 22 Gy, the average decrease in ^99m^TcO_4_^−^ uptake was 3.95%, while in animals that received more than 22 Gy it was 17.5%.

### Kinetics of ^99m^TcO_4_^−^-mediated thyroid stunning

**Figure 3:**
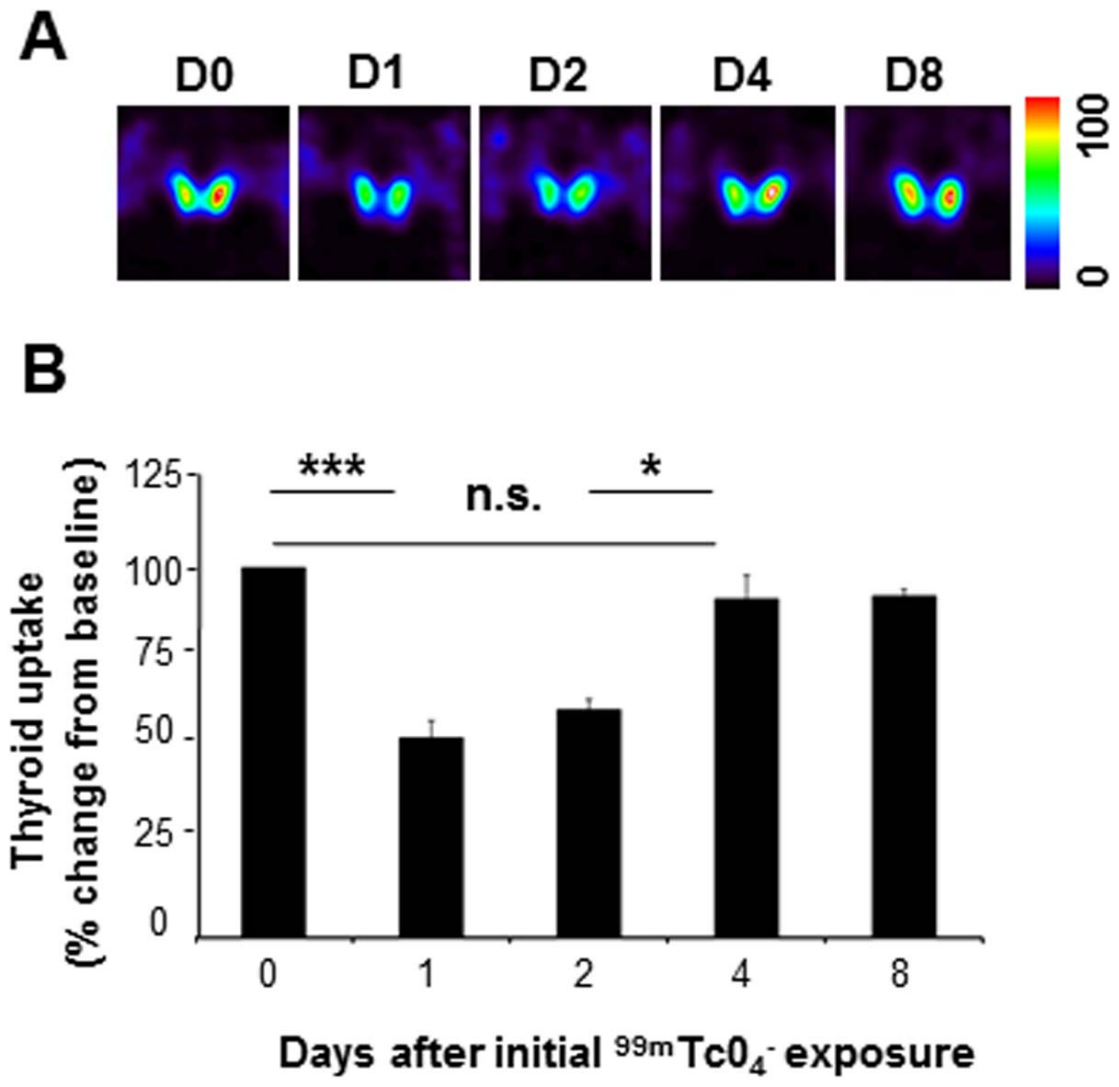
Longitudinal follow up of ^99m^TcO_4_^−^-mediated thyroid stunning by SPECT/CT. ^99m^TcO_4_^−^ uptake in the thyroid was measured by SPECT/CT in 12 mice at day 0 (D0) and again at day 1 (D1, n = 3), day 2 (D2, n = 3), day 4 (D4, n = 3), or day 8 (D8, n = 3). The second scan was performed by injecting a dose of 50 MBq ^99m^TcO_4_^−^. (A) Representative SPECT/CT images of ^99m^TcO_4_^−^ uptake by the thyroid. The images are normalized (100%) to the voxel with the highest activity in the series. (B) Thyroid uptake presented as percentage of the value at day 0± SD. (*: p<0.05; ***: p<0.001).

Using longitudinal follow up of individual mouse thyroids by SPECT/CT imaging, we next determined whether the ^99m^TcO_4_^−^-mediated stunning was reversible. Thyroid activity was measured at day 0, upon administration of 150 MBq of ^99m^TcO_4_^−^, resulting in an absorbed dose to the thyroid of 30 Gy, and reassessed after reinjection of ^99m^TcO_4_^−^, at either day 1, 2, 4, or 8. Figure 3 shows that a single 30 Gy irradiation of the thyroid reduced the magnitude of ^99m^TcO_4_^−^ thyroid uptake by 45% at 24 hours. This impairment in thyroid uptake was observed for up to two days and the ability of the gland to take up the radiotracer was completely restored four days after the initial administration of ^99m^TcO_4_^−^. These data demonstrate that the stunning is rapidly reversible.

### Effect of ^99m^Tc exposure on NIS protein- and mRNA expression

**Figure 4:**
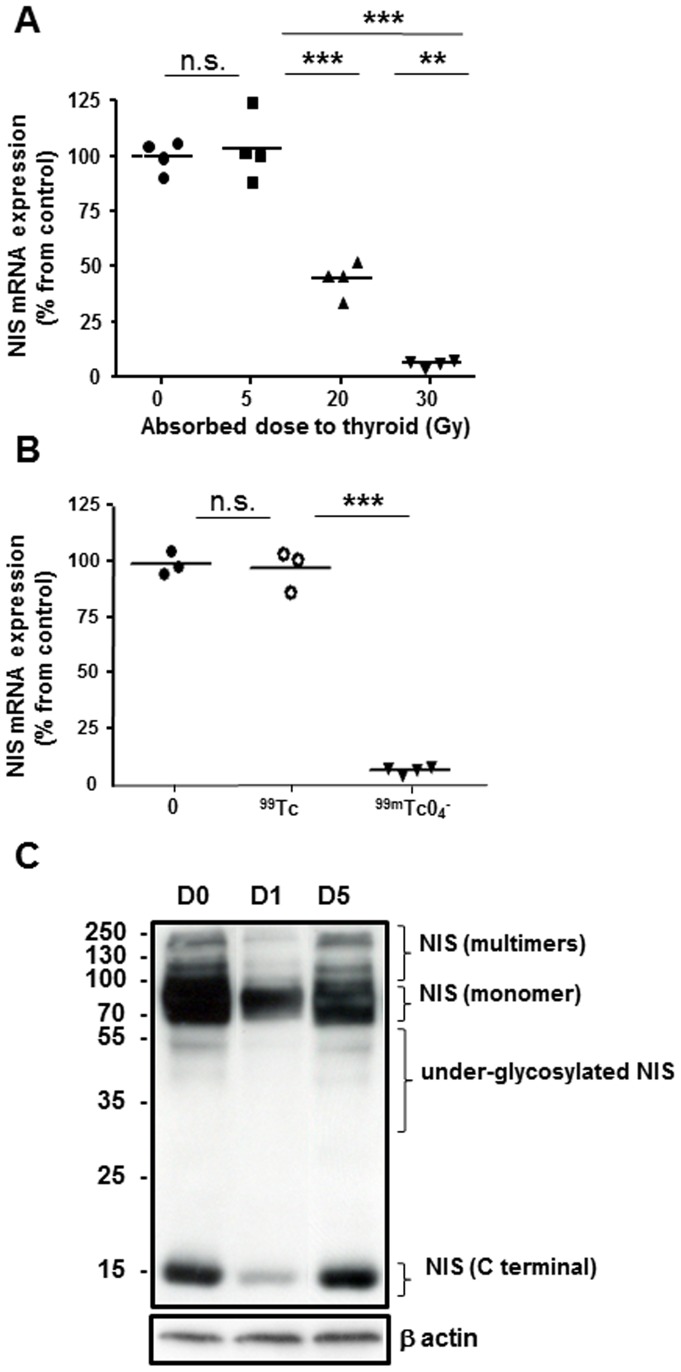
Expression of NIS mRNA and protein in thyroids of mice 24^99m^TcO_4_^−^. (A) Quantitative RT-PCR analyses of NIS expression in control thyroids or thyroids exposed to 5 Gy, 20 Gy or 30 Gy. The means (n = 4 mice/group) are presented (horizontal bars) as well as each individual point. (**: p<0.01; ***: p<0.001). (B) Quantitative RT-PCR analyses of NIS expression in control thyroids or thyroids exposed to decayed ^99m^TcO_4_^−^ (^99^Tc on the figure) or 30 Gy of ^99m^TcO_4_^−^ (^99m^TcO_4_^−^ on the figure). The means (n = 3 to 4 mice/group) are presented (horizontal bars) as well as each individual point. (C) Western blot analyses of NIS expression levels assessed on membrane preparations from thyroids of control mice or from thyroids extracted 1 and 5 days after exposure to 30 Gy ^99m^TcO_4_^−^. The immunoblot shown is representative of three independent experiments.

NIS mRNA levels in mouse thyroid glands were measured 24 hours after a single exposure to ^99m^TcO_4_^−^ (Figure 4A). Expression was high in untreated thyroids but was downregulated by 2.5-fold (P = 0.0007) and 12-fold (P = 0.003) after exposure to 20 Gy and 30 Gy, respectively. In contrast, absorbed doses of 5 Gy failed to induce any significant reduction in the messenger levels. In addition, no marked variation was measured in the mRNA levels of the housekeeping gene, β-actin, measured in the same RNA preparation. To assess whether the reduction in NIS mRNA was associated with the irradiation induced by ^99m^TcO_4_^−^ or to a pharmacological effect of ^99^Tc, we injected mice with a solution of 150 MBq ^99m^TcO_4_^−^ that had been left to decay for three days (about 12 periods). In these conditions, we estimate that the activity of ^99m^TcO_4_^−^ injected is 0.04 MBq, leading to an 8mGy irradiation of the thyroid. In these conditions, most of the technetium is in the form of ^99^Tc. Although ^99^Tc is radioactive, its half-life is very long (211000) years and we can consider that during the course of the experiment (1 day) the irradiation from ^99^Tc is negligible. As expected, injection of a three-days decayed solution of ^99m^TcO_4_^−^ failed to affect the level of NIS mRNA (Figure 4B), suggesting that the stunning induced by ^99m^TcO_4_^−^ is dependent on irradiation.

Western blot analyses of NIS protein expression were performed using membrane preparations from mouse thyroids, 24 hours after a single exposure to 30 Gy ^99m^TcO_4_^−^. Figure 4C shows a marked reduction in the amount of all functional NIS forms, including the higher molecular weight species likely to correspond to multimers and dimers as well as the monomers (90 kDa), compared with control tissues. Although not functional by itself, the 15 kDa band corresponding to the small C-terminal fragment of mNIS [35] was also markedly reduced. Re-expression of all functional NIS isoforms was observed within 5 days following the initial administration of ^99m^TcO_4_^−^, thus demonstrating that the stunning is rapidly reversible (Figure 4C).

### Histologic analysis of ^99m^TcO_4_^−^-exposed thyroids

**Figure 5:**
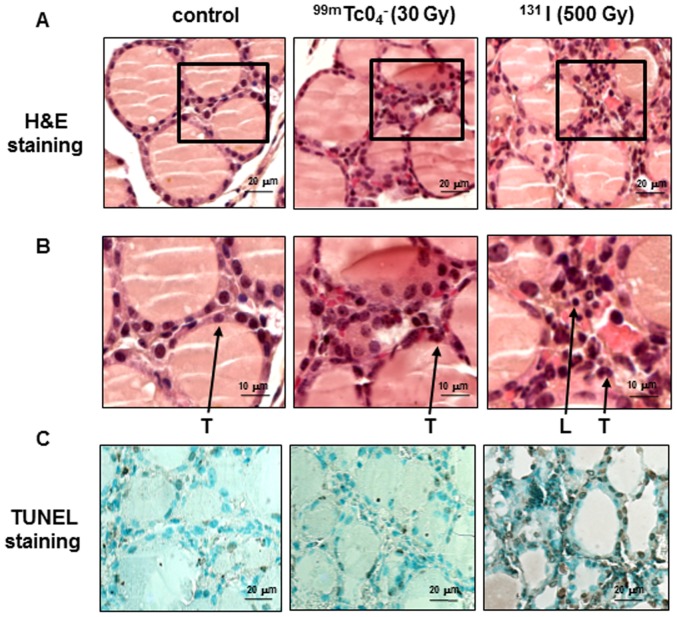
Histology of mouse thyroids 24^99m^TcO_4_^−^ or ^131^I-iodide. (A) Hematoxylin/eosin-stained sections of thyroids show intact morphology after exposure to ^99m^TcO_4_^−^ (30 Gy), but microscopic histological alterations in ^131^I (500 Gy)–exposed thyroids. The squares in the different sections represent the magnified areas presented in (B). (B) Close-up views of the H&E sections presented in (A). L: nuclei of leukocytes, T: nuclei of thyrocytes. (C) Representative apoptosis TUNEL staining of thyroid sections shows rare immunoreactive cells in control and ^99m^Tc-injected mice, but numerous TUNEL-positive nuclei within the thyroids of ^131^I-injected mice.

Hematoxylin-eosin–stained sections of ^99m^TcO_4_^−^-exposed thyroids (absorbed dose of 30 Gy) showed normal thyroid histology, with intact colloid-containing follicles comparable with those of control mice, with no microscopic signs of cytotoxicity (Figure 5A). As a positive control, administration of a therapeutic dose of ^131^I (500 Gy to the thyroid for 24 hours) was used. In this latter condition, the interstitium appears to be expanded by the infiltration of inflammatory cells not present in control or ^99m^TcO_4_^−^-exposed thyroids (Figures 5B). To detect whether these exposures were associated with apoptosis, TUNEL staining was performed. Figure 5C shows a very similar situation in control thyroids and thyroids from ^99m^TcO_4_^−^-exposed animals. By contrast, a strong nuclear TUNEL staining is observed on thyroid sections obtained from mice injected with 500 Gy ^131^I (Figure 5C). More specifically, analysis of 10 randomly-selected fields showed 8 TUNEL-positive nuclei in control thyroids, 9 TUNEL-positive nuclei in ^99m^TcO_4_^−^-exposed thyroids and more than 100 TUNEL-positive nuclei in^131^I-exposed thyroids. These results, combined with the observation that ^99m^TcO_4_^−^-mediated thyroid stunning is recovered within five days of the initial exposure to the radioelement, strongly suggest an absence of significant destruction of the thyroid in response to ^99m^TcO_4_^−^ exposure.

### Induction of double-strand DNA break repair in ^99m^TcO_4_^−^-exposed thyroids

**Figure 6:**
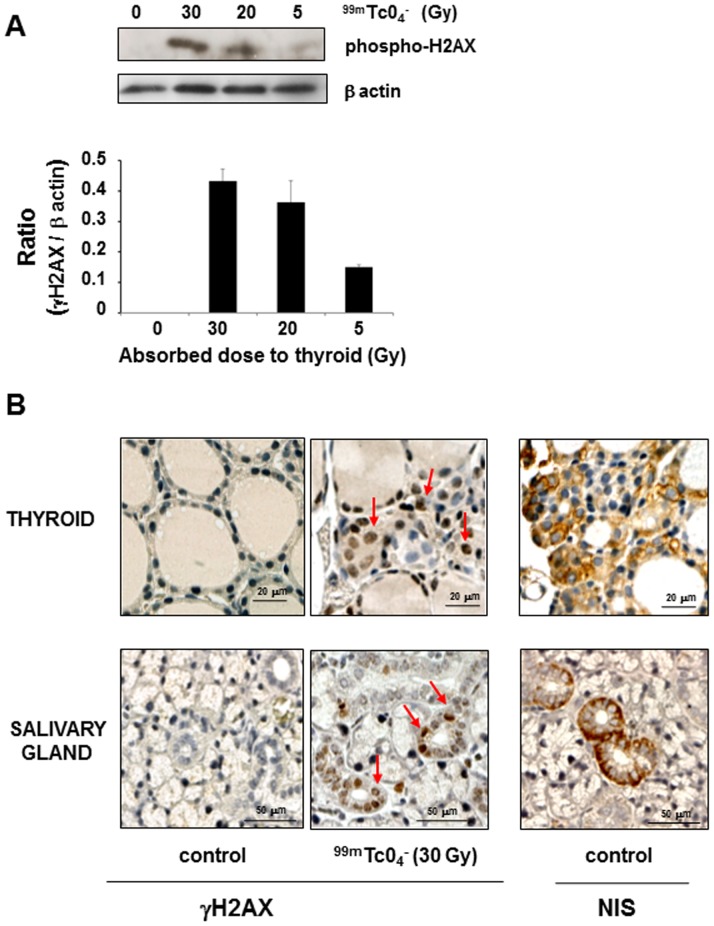
γH2AX foci in thyroids and salivary glands of mice 24 hours after administration of ^99m^TcO_4_^−^. Mice were injected with various activities of ^99m^TcO_4_^−^ and culled 24 hours later for biopsy collection. (A) Western blotting of protein homogenates obtained from mouse thyroids subjected to different levels of irradiation. Results represent the normalized increase in H2AX phosphorylation level compared with the control condition. Protein quantification was performed with image J software. (B) Representative sections of thyroids or salivary glands of control mice or mice exposed to 30 Gy ^99m^TcO_4_^−^ were stained with a γH2AX- or NIS-specific antibody. Arrows represent foci of DNA double-strand break repair (γH2AX-positive nuclei). The figures presented are representative of 2 and 6 independent experiments for Western blot (A) and immunohistochemistry (B), respectively.

We next assessed whether exposure to ^99m^TcO_4_^−^ could lead to the induction of double-strand DNA break repair within the thyroid by following the level of phosphorylation of histone-2AX (γH2AX) [40]. A dose-dependent histone-2AX phosphorylation was detected, by Western blot, on extracts obtained from mouse thyroids subjected to 5, 20 and 30 Gy (Figure 6A). This observation was confirmed by immunohistochemistry (Figure 6B). Foci of nuclear γH2AX immunostaining were visible in sections generated from thyroid sections exposed to 30 Gy. To determine whether the presence of double-strand breaks is dependent on the uptake of the radioisotope by the cells, we next looked at the foci of nuclear γH2AX immunostaining in the salivary glands. In these glands, NIS is selectively expressed in the epithelial cells lining the ducts (Figure 6B) and γH2AX immunostaining was only detected in the nuclei of these ductal cells. This observation strongly suggests that the presence of double-strand breaks is dependent on the uptake of ^99m^TcO_4_^−^ by the cells.

### Global pattern of gene expression in thyroids of ^99m^TcO_4_^−^-exposed versus control mice

**Figure 7:**
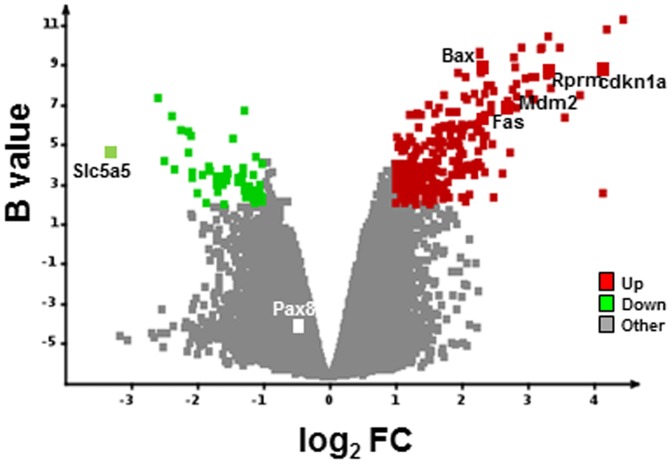
Global pattern of gene expression in thyroids of ^99m^TcO_4_^−^-exposed versus control mice. RNA samples from thyroids of control or ^99m^TcO_4_^−^-exposed mice were harvested (n = 4 per group) 24 hours after radioisotope or saline administration and expression profiles were determined with Agilent arrays. Volcano plot showing the distribution of differentially expressed transcripts between ^99m^TcO_4_^−^-exposed *versus* control thyroids. Log(base2) of the fold-change (LogFC) is plotted against the B-statistic value for each transcript. Several transcripts – NIS/Slc5a5, Pax8, and a subset of p53-regulated genes (*p21/cdkn1a, Rprm, Bax, Mdm2, Fas*) – are highlighted. Significantly down- and upregulated genes are shown in green and red, respectively.

To determine the impact of ^99m^TcO_4_^−^ exposure on the thyroid, total RNA, extracted from four untreated thyroids and four thyroids exposed to 30 Gy with ^99m^TcO_4_^−^, was analyzed using whole genome mouse microarrays. Total RNA was collected 24 hours after radioisotope administration. The comparison between these two conditions showed a significant modulation of 306 transcripts annotated in the RefSeq database, corresponding to 191 up- and 115 downregulated genes in the ^99m^TcO_4_^−^-exposed thyroids (Table S1 and Figure 7). These transcripts efficiently discriminated ^99m^TcO_4_^−^-exposed from control thyroids. As expected from the data presented in Figure 4, NIS (*Slc5a5*) mRNA appeared to be among the most significantly downregulated transcripts in ^99m^TcO_4_^−^-exposed cells, while Pax8, a transcription factor regulating NIS, appeared to be unaffected (Figure 7). Gene ontology analysis using the Mediante information system [39] and Ingenuity Pathway™ showed a very strong association of these modulated transcripts with “p53 signaling” (P = 1.4×10−10) (Table S2). Several genes regulated by p53 were strongly upregulated in 99mTcO4−-exposed thyroids, including cyclin-dependent kinase inhibitor, p21 (p21/Cdkn1a/Waf1/Cip1), reprimo/p53-dependent G2 arrest mediator candidate (Rprm), BCL2-associated X protein (Bax), p53 E3 ubiquitin protein ligase (Mdm2) and members of the TNF receptor family, Fas (Apo-1, Apt1, CD95, Fas1, Fastm, Tnfrsf6) (Table S2).

## Discussion

The first aim of this study was to determine whether ^99m^TcO_4_^−^ was capable of inducing stunning, *in vivo*, in mouse thyroid glands. Our data show that an irradiation above 22 Gy via ^99m^TcO_4_^−^ accumulation in the thyroid leads, in the vast majority of cases, to a marked reduction (up to 40%) in the ability of the thyroid to take up the same radioisotope 24 hours later. This stunning effect, induced by a single injection of ^99m^TcO_4_^−^, could be observed for up to two days, and the ability of the thyroid to accumulate the radiotracer was completely recovered four days after the initial administration of ^99m^TcO_4_^−^. At the molecular level, the initial accumulation of ^99m^TcO_4_^−^ in the thyroid gland led, 24 hours later, to a reduction of NIS mRNA as well as a diminution of all forms of the NIS protein present at the membrane. Histological analysis of thyroid sections did not demonstrate any significant areas of cell death/necrosis in ^99m^TcO_4_^−^-exposed compared with control animals. Altogether, these results are consistent with a stunning effect, defined as a marked diminution of ^99m^TcO_4_^−^ uptake by the thyroid, independent of any significant tissue destruction. The reversibility of this stunning within a few days confirms this hypothesis.

The kinetics of residence of the radioisotope were determined by analysis of SPECT/CT images. Pharmacokinetic data showed that the peak ^99m^TcO_4_^−^ content in the thyroid was reached within 1 hour after intraperitoneal injection and strongly correlated with the cumulated dose at 24 hours. These data are in agreement with the previously published biodistribution of ^99m^TcO_4_^−^ measured by direct counting of radioactivity in biopsies collected at different times from different animals [41]. Our dataset highlights the fact that small-animal SPECT imaging is a very powerful tool, capable of generating reliable data, whilst minimizing the number of experimental animals. Dosimetric calculations revealed that the ^99m^TcO_4_^−^-mediated thyroid stunning effect requires an absorbed dose in the thyroid in the range of 20 Gy, observed in animals injected with an activity of 150 MBq. Activities from 74 to 370 MBq ^99m^TcO_4_^−^ are administered to patients during routine thyroid examination, leading to an absorbed dose to the thyroid below 20 mGy (Society of Nuclear Medicine Procedure Guideline for Thyroid Scintigraphy, http://interactive.snm.org/docs/Thyroid_Scintigraphy_V3.pdf). This large difference in absorbed dose to the thyroid between humans and mice can be explained by the larger volume of distribution of the radioisotope in humans. In addition, all mouse thyrocytes are reported to express NIS, while its expression in human thyrocytes is only patchy [42]. Thus, although ^99m^TcO_4_^−^-mediated thyroid stunning is a real phenomenon in mice, it is very unlikely to be encountered in clinical practice.

The biological events associated/concomitant with ^99m^TcO_4_^−^-mediated stunning were studied by comparing the global pattern of gene expression in the thyroid glands of ^99m^TcO_4_^−^-exposed versus control mice. Several genes were found to be up- or downregulated (see Table S1). Amongst these, the NIS gene (*slc5a5*) was the most highly downregulated. This result is intriguing as the most downregulated gene encodes the protein responsible for the radioisotope uptake, and therefore for the irradiation of the cell. This phenomenon bears the hallmark of an adaptive response of the thyroid to irradiation. Analysis of the whole dataset indicates, with a very high statistical significance (P value in the range of 10^−16^), that the variation in gene expression observed in the thyroids of ^99m^TcO_4_^−^-exposed animals is the result of activation of the p53 pathway. This pathway is typically activated when cells are subjected to stress [43]. In our case, the stress is irradiation of the cells with an absorbed dose in the range of 20 Gy. A similar stress response was reported in thyroids subjected to 11 Gy ^211^At [44]. However, in this latter study NIS gene expression was not reported to vary, suggesting a differential regulation of NIS expression in thyroid cells subjected to α particles and to low-energy electrons.

The ^99m^TcO_4_^−^-mediated irradiation led to the induction of double-strand DNA break repair. Foci of nuclear γH2AX staining were already visible in sections generated from thyroids exposed to 5 Gy, their numbers increased in sections from thyroids exposed to 30 Gy. In the salivary glands, NIS is expressed in ductal epithelial cells, and γH2AX nuclear foci are only detectable in these structures. The salivary gland dataset demonstrates that the biological events triggered by ^99m^TcO_4_^−^ exposure happen selectively in NIS-expressing cells capable of accumulating the radiotracer. The requirement for ^99m^TcO_4_^−^ to enter the cell in order to provoke maximal DNA damage and, if the dose is high enough, cell death, has been demonstrated *in vitro* using the NIS-expressing PCCl3 thyroid cell line [45], [46], [47], [48]. In these experiments, ^99m^TcO_4_^−^-mediated toxicity was clearly magnified by ^99m^TcO_4_^−^ entry into the cells [45], [46], [47] and was further enhanced by ^99m^TcO_4_^−^ retention [48]. This increased radiotoxicity from intracellular ^99m^Tc is likely to be the result of an increased dose deposition in cellular structures due to Auger and conversion electrons, with low range and high local energy deposition properties. The biological effects observed upon internal irradiation of the thyrocytes (double-strand DNA breaks, p53 pathway activation, decrease in NIS protein and mRNA content) may be triggered by a direct or indirect action of low-energy electrons. These mechanisms may not be mutually exclusive and could be involved to different degrees in the different biological events triggered by low-energy electrons. A direct mechanism implies that ^99m^TcO_4_^−^ enters the nucleus and is in direct contact with DNA. Alternatively, following the de-excitation of ^99m^TcO_4_^−^ nuclides in the cell, low-energy electrons may trigger the production of reactive oxygen species (ROS), which could induce the observed biological effects. These ROS could induce DNA damage and activate the p53 pathway. Considering that ROS have already been shown *in vitro* to decrease NIS mRNA levels and reduce iodide uptake [49], ROS may have a preponderant role in ^99m^TcO_4_^−^-mediated thyroid stunning.

In conclusion, we demonstrate in this report that ^99m^TcO_4_^−^-mediated thyroid stunning can be observed *in vivo* in mouse thyroid. This effect is not associated with significant cell death and is reversible within a few days. At the cellular level, decreased NIS mRNA, p53 pathway activation, and the generation of double-strand DNA breaks can be observed. However, considering that the thyroid needs to be exposed to an absorbed dose of at least 20 Gy for a significant stunning, this effect is unlikely to be encountered in clinical practice. Nevertheless, from a biological point of view our system provides a unique experimental setup to compare the sensitivities of different NIS-expressing organs to low-energy electrons and to understand the fine molecular mechanisms involved in cellular stunning.

## Supporting Information

Table S1 **List of the 306 RefSeq annotated transcripts significantly modulated in ^99m^TcO_4_^−^-exposed thyroids.** Agilent Probe and NCBI RefSeq IDs give access to transcript annotations. Logarithm (base 2) of the average intensity and logarithm (base 2) of the ratio ^99m^TcO_4_^−^/control are represented.
(DOCX)

Table S2 **Ingenuity Pathway Analysis of microarray data to highlight selectively affected pathways in ^99m^TcO_4_^−^-exposed thyroids.** Affected canonical pathways ordered by p-value are presented. The probability of obtaining the number of genes in a certain pathway in the list of differentially expressed genes was compared with the representation of the same pathway among all the genes on the microarray; –log10 of the Fisher's exact probability is indicated.
(DOCX)

Material S1 **Dosimetric calculations.**
(DOCX)
