# Supplementary material for: 99mTcO4 −-, Auger-Mediated Thyroid Stunning: Dosimetric Requirements and Associated Molecular Events
Source: PLoS One. 2014 Mar 24;9(3):e92729. doi: 10.1371/journal.pone.0092729 (PMC3963936; doi:10.1371/journal.pone.0092729)
Supplement: Table S1 — List of the 306 RefSeq annotated transcripts significantly modulated in 99mTcO4−-exposed thyroids. Agilent Probe and NCBI RefSeq IDs give access to transcript annotations. Logarithm (base 2) of the average intensity and logarithm (base 2) of the ratio 99mTcO4 −/control are represented. (DOCX) [file pone.0092729.s001.docx]

**Supplemental Table 1: List of the 306 RefSeq annotated transcripts significantly modulated in ^99m^TcO_4_^−^-exposed thyroids.**

| **Gene**  **Name** | **Agilent Probe ID** | **Accession** | **AveExp** | **log_2_ (Tc/Ct)** | **Description** |
| --- | --- | --- | --- | --- | --- |
| Aaas | A_52_P301374 | NM_153416 | 11.06 | 1.69 | achalasia. adrenocortical insufficiency. alacrimia (Aaas) |
| Abhd4 | A_51_P406020 | NM_134076 | 15.50 | 1.15 | abhydrolase domain containing 4 (Abhd4). transcript variant 1 |
| Acer2 | A_55_P2367463 | NM_139306 | 7.64 | 1.03 | alkaline ceramidase 2 (Acer2) |
| Adm2 | A_55_P2181386 | NM_182928 | 7.00 | -1.06 | adrenomedullin 2 (Adm2) |
| Aen | A_55_P2141860 | NM_026531 | 14.42 | 2.00 | apoptosis enhancing nuclease (Aen). transcript variant 1 |
| Alox5 | A_51_P247249 | NM_009662 | 9.69 | 1.58 | arachidonate 5-lipoxygenase (Alox5) |
| Ano1 | A_55_P2038747 | NM_178642 | 12.22 | -1.11 | anoctamin 1. calcium activated chloride channel (Ano1). transcript variant 1 |
| Anxa8 | A_51_P207591 | NM_013473 | 11.21 | 2.10 | annexin A8 (Anxa8) |
| Aox1 | A_51_P333111 | NM_009676 | 13.19 | 1.39 | aldehyde oxidase 1 (Aox1) |
| Apaf1 | A_51_P417701 | NM_001042558 | 8.14 | 1.23 | apoptotic peptidase activating factor 1 (Apaf1). transcript variant 1 |
| Aplnr | A_52_P97572 | NM_011784 | 9.85 | -1.29 | apelin receptor (Aplnr) |
| Aqp6 | A_55_P2182955 | NM_175087 | 11.34 | -1.29 | aquaporin 6 (Aqp6) |
| Arhgef33 | A_66_P107482 | NM_001145452 | 8.71 | -1.61 | Rho guanine nucleotide exchange factor (GEF) 33 (Arhgef33) |
| Arl8a | A_51_P224593 | NM_026823 | 12.51 | 1.01 | ADP-ribosylation factor-like 8A (Arl8a) |
| Armc2 | A_55_P2120469 | NM_001034858 | 10.89 | -1.68 | armadillo repeat containing 2 (Armc2) |
| Asgr1 | A_55_P2003824 | NM_009714 | 13.97 | -1.39 | asialoglycoprotein receptor 1 (Asgr1) |
| Ass1 | A_55_P2143070 | NM_007494 | 11.17 | 3.13 | argininosuccinate synthetase 1 (Ass1) |
| Atp6v0a4 | A_55_P1953003 | NM_080467 | 7.45 | -1.26 | ATPase. H+ transporting. lysosomal V0 subunit A4 (Atp6v0a4) |
| Atp6v1d | A_51_P393598 | NM_023721 | 14.43 | 1.01 | ATPase. H+ transporting. lysosomal V1 subunit D (Atp6v1d) |
| Batf | A_51_P114616 | NM_016767 | 9.62 | -1.15 | basic leucine zipper transcription factor. ATF-like (Batf) |
| Bax | A_55_P2137406 | NM_007527 | 13.21 | 1.95 | BCL2-associated X protein (Bax) |
| Bbc3 | A_51_P248122 | NM_133234 | 12.37 | 1.77 | BCL2 binding component 3 (Bbc3). nuclear gene encoding mitochondrial protein |
| Bcl2a1d | A_55_P1978424 | NM_007536 | 8.71 | -1.43 | B-cell leukemia/lymphoma 2 related protein A1d (Bcl2a1d) |
| Bcl2l1 | A_52_P510877 | NM_009743 | 9.01 | 1.52 | BCL2-like 1 (Bcl2l1). nuclear gene encoding mitochondrial protein |
| Bdh1 | A_55_P2181753 | NM_001122683 | 8.18 | -1.23 | 3-hydroxybutyrate dehydrogenase. type 1 (Bdh1). transcript variant 2 |
| Bloc1s2 | A_55_P1984825 | NM_028607 | 11.68 | 1.01 | biogenesis of lysosome-related organelles complex-1. subunit 2 (Bloc1s2) |
| Bmf | A_55_P2029106 | NM_138313 | 11.21 | -1.37 | BCL2 modifying factor (Bmf) |
| Btg2 | A_52_P31543 | NM_007570 | 11.88 | 1.72 | B-cell translocation gene 2. anti-proliferative (Btg2) |
| Cabp5 | A_55_P2033215 | NM_013877 | 14.64 | 1.30 | calcium binding protein 5 (Cabp5) |
| Cacnb3 | A_51_P259009 | NM_007581 | 9.98 | 1.14 | calcium channel. voltage-dependent. beta 3 subunit (Cacnb3). transcript variant 1 |
| Calml3 | A_55_P2083317 | NM_027416 | 10.33 | 2.49 | calmodulin-like 3 (Calml3) |
| Camkk1 | A_55_P2113165 | NM_018883 | 11.33 | -1.15 | calcium/calmodulin-dependent protein kinase kinase 1. alpha (Camkk1) |
| Capn6 | A_52_P474089 | NM_007603 | 10.99 | -1.25 | calpain 6 (Capn6) |
| Cbs | A_55_P2078670 | NM_144855 | 9.94 | 2.26 | cystathionine beta-synthase (Cbs). transcript variant 1 |
| Ccdc144b | A_55_P2000798 | NM_178418 | 11.57 | 1.67 | coiled-coil domain containing 144B (Ccdc144b) |
| Ccng1 | A_52_P612803 | NM_009831 | 14.03 | 1.78 | cyclin G1 (Ccng1) |
| Ccr5 | A_52_P578732 | NM_009917 | 7.09 | -1.10 | chemokine (C-C motif) receptor 5 (Ccr5) |
| Cd163l1 | A_55_P1989563 | NM_172909 | 7.37 | -1.24 | CD163 molecule-like 1 (Cd163l1) |
| Cd300lf | A_55_P2004511 | NM_001169153 | 10.76 | 1.67 | CD300 antigen like family member F (Cd300lf). transcript variant 1 |
| Cd44 | A_55_P2166488 | NM_009851 | 9.77 | 1.52 | CD44 antigen (Cd44). transcript variant 1 |
| Cd86 | A_55_P1971951 | NM_019388 | 7.70 | -1.12 | CD86 antigen (Cd86) |
| Cdc42bpg | A_52_P105765 | NM_001033342 | 10.14 | 1.53 | CDC42 binding protein kinase gamma (DMPK-like) (Cdc42bpg) |
| Cdca7 | A_55_P2012498 | NM_025866 | 10.91 | -1.59 | cell division cycle associated 7 (Cdca7) |
| Cdh16 | A_51_P287100 | NM_007663 | 14.81 | -1.19 | cadherin 16 (Cdh16). transcript variant 1 |
| Cdkn1a | A_51_P363947 | NM_007669 | 13.02 | 3.87 | cyclin-dependent kinase inhibitor 1A (P21) (Cdkn1a). transcript variant 1 |
| Celf5 | A_55_P1972948 | NM_176954 | 9.91 | 3.31 | CUGBP. Elav-like family member 5 (Celf5) |
| Ces2c | A_55_P2005213 | NM_145603 | 10.30 | 1.71 | carboxylesterase 2C (Ces2c) |
| Ces2e | A_55_P1959500 | NM_172759 | 12.82 | 1.68 | carboxylesterase 2E (Ces2e). transcript variant 1 |
| Cgref1 | A_51_P372550 | NM_026770 | 10.94 | 2.81 | cell growth regulator with EF hand domain 1 (Cgref1). transcript variant 1 |
| Chst3 | A_52_P390944 | NM_016803 | 9.13 | -1.70 | carbohydrate (chondroitin 6/keratan) sulfotransferase 3 (Chst3) |
| Cited4 | A_52_P426768 | NM_019563 | 13.56 | 1.09 | Cbp/p300-interacting transactivator. with Glu/Asp-rich carboxy-terminal domain. 4 (Cited4) |
| Ckap2 | A_52_P162099 | NM_001004140 | 8.53 | 2.36 | cytoskeleton associated protein 2 (Ckap2) |
| Cks1b | A_55_P2061495 | NM_016904 | 10.79 | -1.01 | CDC28 protein kinase 1b (Cks1b) |
| Clec9a | A_66_P103027 | NM_001205363 | 7.04 | -1.15 | C-type lectin domain family 9. member a (Clec9a). transcript variant 1 |
| Cmtm7 | A_55_P1973254 | NM_133978 | 10.84 | -1.25 | CKLF-like MARVEL transmembrane domain containing 7 (Cmtm7). transcript variant 1 |
| Cngb1 | A_55_P2100739 | NM_145601 | 9.46 | -1.14 | cyclic nucleotide gated channel beta 1 (Cngb1). transcript variant 2 |
| Col5a3 | A_51_P241995 | NM_016919 | 11.32 | -1.02 | collagen. type V. alpha 3 (Col5a3) |
| Cox6b2 | A_51_P300506 | NM_183405 | 12.51 | 4.20 | cytochrome c oxidase subunit VIb polypeptide 2 (Cox6b2). transcript variant 1 |
| Cpt1c | A_52_P269942 | NM_153679 | 10.08 | 2.96 | carnitine palmitoyltransferase 1c (Cpt1c). transcript variant 1 |
| Ctxn1 | A_52_P680870 | NM_183315 | 9.97 | 1.31 | cortexin 1 (Ctxn1) |
| Cx3cr1 | A_52_P99810 | NM_009987 | 8.13 | -2.25 | chemokine (C-X3-C) receptor 1 (Cx3cr1) |
| Cxcl12 | A_55_P1966204 | NM_021704 | 10.62 | -1.32 | chemokine (C-X-C motif) ligand 12 (Cxcl12). transcript variant 1 |
| Cxcr6 | A_55_P2165199 | NM_030712 | 8.06 | -1.28 | chemokine (C-X-C motif) receptor 6 (Cxcr6) |
| Cyp27b1 | A_55_P2088237 | NM_010009 | 7.25 | -1.56 | cytochrome P450. family 27. subfamily b. polypeptide 1 (Cyp27b1). nuclear gene encoding mitochondrial protein |
| Daf2 | A_66_P106385 | NM_007827 | 9.53 | 1.31 | decay accelerating factor 2 (Daf2) |
| Dcaf12l2 | A_55_P1959973 | NM_175539 | 12.84 | 1.93 | DDB1 and CUL4 associated factor 12-like 2 (Dcaf12l2) |
| Dcaf4 | A_51_P170641 | NM_030246 | 11.18 | 1.15 | DDB1 and CUL4 associated factor 4 (Dcaf4). transcript variant 2 |
| Dcbld1 | A_55_P1967905 | NM_025705 | 9.71 | 1.01 | discoidin. CUB and LCCL domain containing 1 (Dcbld1) |
| Dclk3 | A_51_P419637 | NM_172928 | 9.53 | -1.81 | doublecortin-like kinase 3 (Dclk3) |
| Dcxr | A_51_P181319 | NM_026428 | 12.26 | 2.91 | dicarbonyl L-xylulose reductase (Dcxr) |
| Ddit4 | A_51_P245796 | NM_029083 | 14.24 | 2.28 | DNA-damage-inducible transcript 4 (Ddit4) |
| Ddx25 | A_51_P326229 | NM_013932 | 10.45 | 2.63 | DEAD (Asp-Glu-Ala-Asp) box polypeptide 25 (Ddx25) |
| Dio1 | A_51_P403477 | NM_007860 | 12.93 | -2.34 | deiodinase. iodothyronine. type I (Dio1) |
| Dnaja1 | A_55_P2087984 | NM_001164671 | 14.50 | 1.04 | DnaJ (Hsp40) homolog. subfamily A. member 1 (Dnaja1). transcript variant 1 |
| Dnali1 | A_55_P2040723 | NM_175223 | 8.14 | 1.21 | dynein. axonemal. light intermediate polypeptide 1 (Dnali1) |
| Dnm3 | A_52_P164286 | NM_001038619 | 8.10 | 1.58 | dynamin 3 (Dnm3). transcript variant 1 |
| Dos | A_51_P436817 | NM_001195268 | 11.87 | 1.27 | downstream of Stk11 (Dos). transcript variant 1 |
| Dtx4 | A_51_P144349 | NM_172442 | 10.95 | -1.21 | deltex 4 homolog (Drosophila) (Dtx4) |
| Duoxa1 | A_55_P2046443 | NM_145395 | 12.32 | 2.29 | dual oxidase maturation factor 1 (Duoxa1) |
| Duoxa2 | A_55_P2006644 | NM_025777 | 9.50 | -1.80 | dual oxidase maturation factor 2 (Duoxa2) |
| Dyrk3 | A_52_P232580 | NM_145508 | 10.47 | 2.10 | dual-specificity tyrosine-(Y)-phosphorylation regulated kinase 3 (Dyrk3) |
| Egfl6 | A_52_P655687 | NM_019397 | 7.24 | -1.55 | EGF-like-domain. multiple 6 (Egfl6) |
| Ei24 | A_55_P2175284 | NM_001199494 | 10.79 | 1.42 | etoposide induced 2.4 mRNA (Ei24). transcript variant 1 |
| Emid1 | A_55_P1954277 | NM_080595 | 10.81 | -1.15 | EMI domain containing 1 (Emid1) |
| Enc1 | A_51_P126437 | NM_007930 | 11.40 | 1.01 | ectodermal-neural cortex 1 (Enc1) |
| Epha2 | A_52_P518997 | NM_010139 | 11.32 | 1.84 | Eph receptor A2 (Epha2) |
| Ephx1 | A_55_P2002578 | NM_010145 | 16.41 | 1.94 | epoxide hydrolase 1. microsomal (Ephx1) |
| Eppk1 | A_55_P2110351 | NM_144848 | 12.74 | 3.03 | epiplakin 1 (Eppk1) |
| Ercc5 | A_66_P126189 | NM_011729 | 10.24 | 1.20 | excision repair cross-complementing rodent repair deficiency. complementation group 5 (Ercc5) |
| Esrrb | A_55_P1954302 | NM_001159500 | 11.77 | -1.81 | estrogen related receptor. beta (Esrrb). transcript variant 2 |
| Evi2a | A_55_P2017759 | NM_001033711 | 8.31 | -1.61 | ecotropic viral integration site 2a (Evi2a). transcript variant 1 |
| Exoc4 | A_51_P350403 | NM_009148 | 10.33 | 1.23 | exocyst complex component 4 (Exoc4) |
| Fas | A_55_P2091676 | NM_007987 | 11.89 | 1.93 | Fas (TNF receptor superfamily member 6) (Fas). transcript variant 1 |
| Fat1 | A_55_P2107502 | NM_001081286 | 13.54 | 1.32 | FAT tumor suppressor homolog 1 (Drosophila) (Fat1) |
| Fbxw9 | A_51_P357561 | NM_026791 | 10.47 | 1.12 | F-box and WD-40 domain protein 9 (Fbxw9) |
| Foxj1 | A_51_P456870 | NM_008240 | 8.56 | 2.10 | forkhead box J1 (Foxj1) |
| Gadd45g | A_51_P315904 | NM_011817 | 14.44 | 1.55 | growth arrest and DNA-damage-inducible 45 gamma (Gadd45g) |
| Galnt5 | A_55_P2122871 | NM_172855 | 7.68 | -1.32 | UDP-N-acetyl-alpha-D-galactosamine:polypeptide N-acetylgalactosaminyltransferase 5 (Galnt5) |
| Gas6 | A_51_P172054 | NM_019521 | 15.86 | 1.32 | growth arrest specific 6 (Gas6) |
| Gdf15 | A_55_P1960735 | NM_011819 | 14.84 | 4.15 | growth differentiation factor 15 (Gdf15) |
| Gfer | A_55_P2144796 | NM_023040 | 11.05 | 1.22 | growth factor. erv1 (S. cerevisiae)-like (augmenter of liver regeneration) (Gfer) |
| Gjb3 | A_55_P2014124 | NM_001160012 | 9.01 | -1.38 | gap junction protein. beta 3 (Gjb3). transcript variant 1 |
| Gmnn | A_55_P2128646 | NM_020567 | 9.56 | -1.34 | geminin (Gmnn) |
| Gpr34 | A_55_P2039684 | NM_011823 | 7.50 | -1.29 | G protein-coupled receptor 34 (Gpr34) |
| Gpr65 | A_51_P108459 | NM_008152 | 7.66 | -1.69 | G-protein coupled receptor 65 (Gpr65) |
| Gramd2 | A_55_P1970901 | NM_001033498 | 9.05 | 1.07 | GRAM domain containing 2 (Gramd2) |
| Gria3 | A_52_P68221 | NM_016886 | 8.43 | 1.59 | glutamate receptor. ionotropic. AMPA3 (alpha 3) (Gria3) |
| Grp | A_51_P356055 | NM_175012 | 10.86 | -2.09 | gastrin releasing peptide (Grp) |
| Gtse1 | A_51_P195153 | NM_013882 | 8.13 | 3.30 | G two S phase expressed protein 1 (Gtse1). transcript variant 2 |
| H2-Aa | A_52_P343306 | NM_010378 | 8.37 | -1.68 | histocompatibility 2. class II antigen A. alpha (H2-Aa) |
| H2-Ab1 | A_55_P1962747 | NM_207105 | 14.02 | -1.81 | histocompatibility 2. class II antigen A. beta 1 (H2-Ab1) |
| H2afj | A_55_P2099540 | NM_177688 | 11.06 | 1.55 | H2A histone family. member J (H2afj) |
| H2-Ea-ps | A_51_P222741 | NM_010381 | 7.76 | -1.35 | histocompatibility 2. class II antigen E alpha. pseudogene (H2-Ea-ps) |
| H2-Eb1 | A_55_P2156731 | NM_010382 | 13.91 | -1.71 | histocompatibility 2. class II antigen E beta (H2-Eb1) |
| Hbegf | A_51_P181565 | NM_010415 | 11.23 | 1.32 | heparin-binding EGF-like growth factor (Hbegf) |
| Herc2 | A_55_P2106429 | NM_010418 | 12.37 | 1.04 | hect (homologous to the E6-AP (UBE3A) carboxyl terminus) domain and RCC1 (CHC1)-like domain (RLD) 2 (Herc2) |
| Heyl | A_52_P337259 | NM_013905 | 9.63 | -1.45 | hairy/enhancer-of-split related with YRPW motif-like (Heyl) |
| Hist1h2be | A_55_P2109128 | NM_178194 | 9.68 | 1.07 | histone cluster 1. H2be (Hist1h2be). transcript variant 2 |
| Hist2h4 | A_55_P1974967 | NM_033596 | 7.44 | 1.10 | histone cluster 2. H4 (Hist2h4) |
| Hp | A_55_P2048607 | NM_017370 | 14.56 | 1.16 | haptoglobin (Hp) |
| Hpgd | A_51_P458778 | NM_008278 | 7.82 | -1.27 | hydroxyprostaglandin dehydrogenase 15 (NAD) (Hpgd) |
| Hspa1a | A_55_P2068459 | NM_010479 | 11.71 | 1.60 | heat shock protein 1A (Hspa1a) |
| Hunk | A_55_P1967538 | NM_015755 | 8.71 | -1.74 | hormonally upregulated Neu-associated kinase (Hunk) |
| Hyal1 | A_51_P479408 | NM_008317 | 10.51 | 1.09 | hyaluronoglucosaminidase 1 (Hyal1) |
| Il20ra | A_51_P340170 | NM_172786 | 8.61 | 1.48 | interleukin 20 receptor. alpha (Il20ra) |
| Inf2 | A_55_P2085295 | NM_198411 | 13.96 | 1.67 | inverted formin. FH2 and WH2 domain containing (Inf2) |
| Inmt | A_51_P162162 | NM_009349 | 11.32 | -1.44 | indolethylamine N-methyltransferase (Inmt) |
| Inpp5j | A_55_P2115871 | NM_172439 | 10.72 | -1.10 | inositol polyphosphate 5-phosphatase J (Inpp5j) |
| Ipcef1 | A_51_P438657 | NM_001170800 | 7.07 | -1.29 | interaction protein for cytohesin exchange factors 1 (Ipcef1). transcript variant 1 |
| Irf8 | A_52_P354823 | NM_008320 | 11.00 | -1.24 | interferon regulatory factor 8 (Irf8) |
| Itga10 | A_55_P2111533 | NM_001081053 | 9.06 | 1.21 | integrin. alpha 10 (Itga10) |
| Itgax | A_51_P303424 | NM_021334 | 8.19 | -1.69 | integrin alpha X (Itgax) |
| Itih4 | A_55_P2077055 | NM_018746 | 8.88 | 1.14 | inter alpha-trypsin inhibitor. heavy chain 4 (Itih4). transcript variant 1 |
| Jak3 | A_55_P2010912 | NM_001190830 | 9.39 | 1.02 | Janus kinase 3 (Jak3). transcript variant 2 |
| Kcnj4 | A_51_P166740 | NM_008427 | 7.24 | 1.60 | potassium inwardly-rectifying channel. subfamily J. member 4 (Kcnj4) |
| Kcnn2 | A_51_P309854 | NM_080465 | 8.69 | -2.01 | potassium intermediate/small conductance calcium-activated channel. subfamily N. member 2 (Kcnn2) |
| Kit | A_66_P128434 | NM_001122733 | 10.59 | -1.10 | kit oncogene (Kit). transcript variant 1 |
| Klhl14 | A_55_P2048085 | NM_001081403 | 9.93 | -1.58 | kelch-like 14 (Drosophila) (Klhl14) |
| Klhl26 | A_51_P143468 | NM_178771 | 11.61 | 1.11 | kelch-like 26 (Drosophila) (Klhl26). transcript variant 2 |
| Klra1 | A_55_P2004551 | NM_016659 | 9.47 | 1.51 | killer cell lectin-like receptor. subfamily A. member 1 (Klra1) |
| Krt73 | A_55_P1973970 | NM_212485 | 9.91 | 1.30 | keratin 73 (Krt73) |
| Lace1 | A_51_P432511 | NM_145743 | 10.27 | 1.03 | lactation elevated 1 (Lace1) |
| Lama5 | A_55_P2064321 | NM_001081171 | 13.62 | 1.23 | laminin. alpha 5 (Lama5) |
| Lass6 | A_52_P667287 | NM_172856 | 7.82 | -1.29 | LAG1 homolog. ceramide synthase 6 (Lass6) |
| Lcn5 | A_55_P1963727 | NM_007947 | 7.36 | 1.54 | lipocalin 5 (Lcn5). transcript variant 1 |
| Lcn8 | A_55_P2051738 | NM_033145 | 9.17 | 3.35 | lipocalin 8 (Lcn8) |
| Lgi3 | A_51_P356967 | NM_145219 | 9.09 | -1.41 | leucine-rich repeat LGI family. member 3 (Lgi3) |
| Lifr | A_55_P2159264 | NM_001113386 | 10.19 | -1.17 | leukemia inhibitory factor receptor (Lifr). transcript variant 2 |
| Lrfn4 | A_55_P2138878 | NM_153388 | 10.32 | -1.22 | leucine rich repeat and fibronectin type III domain containing 4 (Lrfn4) |
| Ltbp2 | A_55_P1970105 | NM_013589 | 13.24 | 1.05 | latent transforming growth factor beta binding protein 2 (Ltbp2) |
| Ly86 | A_51_P465350 | NM_010745 | 9.34 | -1.30 | lymphocyte antigen 86 (Ly86) |
| Lypd6b | A_51_P105068 | NM_027990 | 11.42 | -1.20 | LY6/PLAUR domain containing 6B (Lypd6b) |
| Mab21l3 | A_55_P2090060 | NM_172295 | 8.89 | 2.26 | mab-21-like 3 (C. elegans) (Mab21l3) |
| Mapk4 | A_51_P245895 | NM_172632 | 7.34 | -1.01 | mitogen-activated protein kinase 4 (Mapk4) |
| Mapre3 | A_55_P2087404 | NM_133350 | 13.53 | 1.12 | microtubule-associated protein. RP/EB family. member 3 (Mapre3) |
| Matn4 | A_55_P2171206 | NM_013592 | 10.77 | -1.16 | matrilin 4 (Matn4). transcript variant 1 |
| Mbnl3 | A_55_P2106255 | NM_134163 | 7.88 | -1.17 | muscleblind-like 3 (Drosophila) (Mbnl3) |
| Mc4r | A_55_P2183884 | NM_016977 | 8.22 | -1.20 | melanocortin 4 receptor (Mc4r) |
| Mdm2 | A_55_P1975475 | NM_010786 | 14.07 | 2.35 | transformed mouse 3T3 cell double minute 2 (Mdm2) |
| Mdm4 | A_52_P485905 | NM_008575 | 8.65 | 1.65 | transformed mouse 3T3 cell double minute 4 (Mdm4) |
| Mfi2 | A_51_P324351 | NM_013900 | 9.19 | -2.08 | antigen p97 (melanoma associated) identified by monoclonal antibodies 133.2 and 96.5 (Mfi2) |
| Mfsd2a | A_51_P279437 | NM_029662 | 9.02 | 2.23 | major facilitator superfamily domain containing 2A (Mfsd2a) |
| Mgmt | A_55_P2071858 | NM_008598 | 11.93 | 2.27 | O-6-methylguanine-DNA methyltransferase (Mgmt) |
| Micall1 | A_51_P242414 | NM_177461 | 12.50 | 1.12 | microtubule associated monoxygenase. calponin and LIM domain containing -like 1 (Micall1) |
| Mpeg1 | A_51_P390538 | NM_010821 | 10.99 | -1.23 | macrophage expressed gene 1 (Mpeg1) |
| Mrps6 | A_52_P58257 | NM_080456 | 14.77 | 1.01 | mitochondrial ribosomal protein S6 (Mrps6). nuclear gene encoding mitochondrial protein |
| Ms4a10 | A_55_P2094445 | NM_023529 | 8.15 | 2.30 | membrane-spanning 4-domains. subfamily A. member 10 (Ms4a10) |
| Mybl1 | A_55_P1966369 | NM_008651 | 7.29 | 1.39 | myeloblastosis oncogene-like 1 (Mybl1) |
| Ncs1 | A_51_P114062 | NM_019681 | 9.62 | -1.02 | neuronal calcium sensor 1 (Ncs1) |
| Net1 | A_66_P105736 | NM_019671 | 12.01 | 1.73 | neuroepithelial cell transforming gene 1 (Net1). transcript variant 1 |
| Neu3 | A_52_P357611 | NM_016720 | 9.61 | 1.93 | neuraminidase 3 (Neu3) |
| Nkx2-9 | A_55_P2042844 | NM_008701 | 8.52 | 3.56 | NK2 transcription factor related. locus 9 (Drosophila) (Nkx2-9) |
| Nme5 | A_55_P1966102 | NM_080637 | 11.42 | 1.12 | non-metastatic cells 5. protein expressed in (nucleoside-diphosphate kinase) (Nme5) |
| Nmur2 | A_55_P2179582 | NM_153079 | 10.55 | 1.48 | neuromedin U receptor 2 (Nmur2) |
| Notch3 | A_51_P220162 | NM_008716 | 13.03 | 1.06 | Notch gene homolog 3 (Drosophila) (Notch3) |
| Nrg1 | A_55_P1985433 | NM_178591 | 11.11 | 1.83 | neuregulin 1 (Nrg1) |
| Nudt10 | A_55_P1993807 | NM_001031664 | 9.51 | -1.00 | nudix (nucleoside diphosphate linked moiety X)-type motif 10 (Nudt10) |
| Odz4 | A_55_P2141084 | NM_011858 | 14.10 | -1.30 | odd Oz/ten-m homolog 4 (Drosophila) (Odz4) |
| Olfr1229 | A_55_P2142172 | NM_001011761 | 10.48 | 1.46 | olfactory receptor 1229 (Olfr1229) |
| Papss2 | A_55_P2080021 | NM_011864 | 14.29 | -1.25 | 3'-phosphoadenosine 5'-phosphosulfate synthase 2 (Papss2). transcript variant 1 |
| Pdgfra | A_51_P345649 | NM_011058 | 9.81 | -1.07 | platelet derived growth factor receptor. alpha polypeptide (Pdgfra). transcript variant 1 |
| Pdzk1ip1 | A_55_P2011678 | NM_001164557 | 11.67 | -1.15 | PDZK1 interacting protein 1 (Pdzk1ip1). transcript variant 1 |
| Per1 | A_55_P1970033 | NM_011065 | 12.68 | 2.20 | period homolog 1 (Drosophila) (Per1). transcript variant 1 |
| Per2 | A_51_P282760 | NM_011066 | 9.62 | 2.04 | period homolog 2 (Drosophila) (Per2) |
| Perp | A_51_P317941 | NM_022032 | 14.12 | 2.01 | PERP. TP53 apoptosis effector (Perp) |
| Phlda3 | A_51_P329928 | NM_013750 | 15.82 | 1.41 | pleckstrin homology-like domain. family A. member 3 (Phlda3) |
| Pigf | A_51_P264053 | NM_008838 | 10.93 | 1.12 | phosphatidylinositol glycan anchor biosynthesis. class F (Pigf) |
| Pim3 | A_51_P189746 | NM_145478 | 11.85 | 1.05 | proviral integration site 3 (Pim3) |
| Plcd4 | A_52_P327588 | NM_148937 | 11.75 | 1.97 | phospholipase C. delta 4 (Plcd4). transcript variant 2 |
| Plk2 | A_51_P290576 | NM_152804 | 10.14 | 1.20 | polo-like kinase 2 (Drosophila) (Plk2) |
| Plk3 | A_51_P375201 | NM_013807 | 9.68 | 1.11 | polo-like kinase 3 (Drosophila) (Plk3) |
| Plk5 | A_55_P1972297 | NM_183152 | 8.92 | 2.36 | polo-like kinase 5 (Drosophila) (Plk5) |
| Pm20d1 | A_55_P2159585 | NM_178079 | 10.14 | 1.57 | peptidase M20 domain containing 1 (Pm20d1) |
| Pmaip1 | A_51_P477121 | NM_021451 | 8.67 | 2.73 | phorbol-12-myristate-13-acetate-induced protein 1 (Pmaip1) |
| Polk | A_55_P2000533 | NM_012048 | 10.09 | 2.01 | polymerase (DNA directed). kappa (Polk) |
| Ppp1r13l | A_55_P2074924 | NM_001010836 | 11.63 | 1.93 | protein phosphatase 1. regulatory (inhibitor) subunit 13 like (Ppp1r13l) |
| Ppp1r1b | A_55_P2026275 | NM_144828 | 13.89 | -1.85 | protein phosphatase 1. regulatory (inhibitor) subunit 1B (Ppp1r1b) |
| Pqlc3 | A_51_P332652 | NM_172574 | 11.89 | 1.24 | PQ loop repeat containing (Pqlc3). transcript variant 1 |
| Prdx6 | A_55_P2176731 | NM_007453 | 14.87 | 1.24 | peroxiredoxin 6 (Prdx6) |
| Pros1 | A_51_P393426 | NM_011173 | 11.35 | 1.38 | protein S (alpha) (Pros1) |
| Prosapip1 | A_55_P2020361 | NM_197945 | 14.39 | 1.33 | ProSAPiP1 protein (Prosapip1) |
| Prr15l | A_52_P435561 | NM_146026 | 10.93 | 1.25 | proline rich 15-like (Prr15l) |
| Psapl1 | A_52_P523368 | NM_175249 | 11.37 | 4.16 | prosaposin-like 1 (Psapl1) |
| Psrc1 | A_55_P2429225 | NM_001190161 | 9.34 | 3.49 | proline/serine-rich coiled-coil 1 (Psrc1). transcript variant 1 |
| Ptgir | A_55_P1994309 | NM_008967 | 9.04 | -1.37 | prostaglandin I receptor (IP) (Ptgir) |
| Ptk2b | A_51_P311904 | NM_172498 | 11.70 | 1.98 | PTK2 protein tyrosine kinase 2 beta (Ptk2b). transcript variant 3 |
| Ptpdc1 | A_55_P2007871 | NM_207232 | 12.03 | 1.40 | protein tyrosine phosphatase domain containing 1 (Ptpdc1) |
| Ptpn22 | A_51_P483324 | NM_008979 | 8.17 | -1.71 | protein tyrosine phosphatase. non-receptor type 22 (lymphoid) (Ptpn22) |
| Pvt1 | A_55_P2051254 | NR_003368 | 9.11 | 2.33 | plasmacytoma variant translocation 1 (Pvt1). non-coding RNA |
| Rab3il1 | A_55_P1994927 | NM_144538 | 10.75 | -1.01 | RAB3A interacting protein (rabin3)-like 1 (Rab3il1) |
| Rabgap1 | A_55_P2161923 | NM_001033960 | 10.02 | 1.54 | RAB GTPase activating protein 1 (Rabgap1). transcript variant 2 |
| Rabgap1l | A_55_P2002819 | NM_013862 | 12.52 | 1.11 | RAB GTPase activating protein 1-like (Rabgap1l). transcript variant 1 |
| Rad51ap1 | A_55_P1991688 | NM_009013 | 8.69 | -1.09 | RAD51 associated protein 1 (Rad51ap1) |
| Rasgef1b | A_55_P2035315 | NM_145839 | 10.97 | -1.07 | RasGEF domain family. member 1B (Rasgef1b). transcript variant 1 |
| Rbp1 | A_55_P2059010 | NM_011254 | 13.35 | -1.37 | retinol binding protein 1. cellular (Rbp1) |
| Reep2 | A_52_P125467 | NM_144865 | 8.46 | 1.16 | receptor accessory protein 2 (Reep2). transcript variant 1 |
| Rem2 | A_51_P191909 | NM_080726 | 8.03 | -1.13 | rad and gem related GTP binding protein 2 (Rem2) |
| Rev1 | A_51_P249594 | NM_019570 | 9.65 | 1.11 | REV1 homolog (S. cerevisiae) (Rev1) |
| Rhbdf2 | A_55_P2028259 | NM_172572 | 10.93 | 1.25 | rhomboid 5 homolog 2 (Drosophila) (Rhbdf2). transcript variant 1 |
| Rhpn1 | A_55_P2077027 | NM_001163465 | 10.09 | -1.32 | rhophilin. Rho GTPase binding protein 1 (Rhpn1). transcript variant 1 |
| Rimkla | A_52_P69194 | NM_177572 | 8.65 | -1.53 | ribosomal modification protein rimK-like family member A (Rimkla) |
| Ripk4 | A_55_P2035400 | NM_023663 | 12.69 | 1.47 | receptor-interacting serine-threonine kinase 4 (Ripk4) |
| Rln1 | A_52_P182298 | NM_011272 | 9.21 | 2.30 | relaxin 1 (Rln1) |
| Rnf169 | A_52_P577729 | NM_175388 | 12.11 | 1.61 | ring finger protein 169 (Rnf169) |
| Rnf183 | A_55_P1988310 | NM_153504 | 8.89 | -1.45 | ring finger protein 183 (Rnf183) |
| Robo3 | A_55_P2144886 | NM_001164767 | 7.81 | 1.00 | roundabout homolog 3 (Drosophila) (Robo3) |
| Rprm | A_51_P278653 | NM_023396 | 9.44 | 3.29 | reprimo. TP53 dependent G2 arrest mediator candidate (Rprm) |
| Rps27l | A_51_P246903 | NM_026467 | 15.68 | 1.22 | ribosomal protein S27-like (Rps27l) |
| Rufy4 | A_55_P2031471 | NM_001034060 | 10.33 | 1.00 | RUN and FYVE domain containing 4 (Rufy4). transcript variant 2 |
| S100a3 | A_51_P468456 | NM_011310 | 8.53 | 1.17 | S100 calcium binding protein A3 (S100a3) |
| Scnn1a | A_51_P213691 | NM_011324 | 13.66 | 1.47 | sodium channel. nonvoltage-gated 1 alpha (Scnn1a) |
| Scube2 | A_55_P2232988 | NM_020052 | 10.81 | -1.58 | signal peptide. CUB domain. EGF-like 2 (Scube2) |
| Scx | A_51_P380432 | NM_198885 | 11.60 | -1.69 | scleraxis (Scx) |
| Sdc1 | A_52_P479269 | NM_011519 | 10.00 | 1.37 | syndecan 1 (Sdc1) |
| Sec11c | A_51_P359262 | NM_025468 | 11.94 | -1.01 | SEC11 homolog C (S. cerevisiae) (Sec11c) |
| Serpina3n | A_51_P159453 | NM_009252 | 8.32 | 2.13 | serine (or cysteine) peptidase inhibitor. clade A. member 3N (Serpina3n) |
| Serpinb6b | A_55_P2013043 | NM_011454 | 10.80 | 1.41 | serine (or cysteine) peptidase inhibitor. clade B. member 6b (Serpinb6b) |
| Sesn2 | A_51_P161354 | NM_144907 | 12.20 | 1.84 | sestrin 2 (Sesn2) |
| Sgk2 | A_55_P2042923 | NM_013731 | 7.82 | 1.72 | serum/glucocorticoid regulated kinase 2 (Sgk2) |
| Sh3bgrl2 | A_52_P316933 | NM_172507 | 10.79 | 1.25 | SH3 domain binding glutamic acid-rich protein like 2 (Sh3bgrl2) |
| Shc4 | A_55_P1968763 | NM_199022 | 13.90 | 1.56 | SHC (Src homology 2 domain containing) family. member 4 (Shc4) |
| Siglech | A_55_P2165790 | NM_178706 | 8.15 | -1.45 | sialic acid binding Ig-like lectin H (Siglech) |
| Slain1 | A_51_P147284 | NM_198014 | 9.16 | -1.04 | SLAIN motif family. member 1 (Slain1) |
| Slamf8 | A_51_P444290 | NM_029084 | 7.34 | -1.17 | SLAM family member 8 (Slamf8) |
| Slc10a6 | A_51_P173678 | NM_029415 | 8.04 | 1.25 | solute carrier family 10 (sodium/bile acid cotransporter family). member 6 (Slc10a6) |
| Slc17a9 | A_55_P1998601 | NM_183161 | 8.65 | -1.12 | solute carrier family 17. member 9 (Slc17a9) |
| Slc19a2 | A_51_P329332 | NM_054087 | 10.48 | 2.20 | solute carrier family 19 (thiamine transporter). member 2 (Slc19a2) |
| Slc23a1 | A_52_P141628 | NM_011397 | 11.28 | 3.10 | solute carrier family 23 (nucleobase transporters). member 1 (Slc23a1) |
| Slc26a10 | A_52_P140881 | NM_177615 | 12.53 | -2.58 | solute carrier family 26. member 10 (Slc26a10) |
| Slc27a3 | A_55_P1958597 | NM_011988 | 11.41 | 1.03 | solute carrier family 27 (fatty acid transporter). member 3 (Slc27a3) |
| Slc2a9 | A_55_P2008936 | NM_001102414 | 7.90 | 1.40 | solute carrier family 2 (facilitated glucose transporter). member 9 (Slc2a9). transcript variant 1 |
| Slc44a4 | A_52_P517762 | NM_023557 | 8.20 | -1.25 | solute carrier family 44. member 4 (Slc44a4) |
| Slc5a3 | A_52_P283055 | NM_017391 | 11.57 | 2.06 | solute carrier family 5 (inositol transporters). member 3 (Slc5a3) |
| Slc5a5 | A_51_P115738 | NM_053248 | 10.09 | -3.27 | solute carrier family 5 (sodium iodide symporter). member 5 (Slc5a5) |
| Slco2a1 | A_55_P1989673 | NM_033314 | 12.20 | -1.14 | solute carrier organic anion transporter family. member 2a1 (Slco2a1) |
| Smoc2 | A_55_P2002903 | NM_022315 | 14.43 | -1.24 | SPARC related modular calcium binding 2 (Smoc2) |
| Snhg12 | A_66_P100249 | NR_029468 | 11.71 | 1.02 | small nucleolar RNA host gene 12 (Snhg12). non-coding RNA |
| Socs1 | A_51_P279606 | NM_009896 | 9.72 | -2.12 | suppressor of cytokine signaling 1 (Socs1) |
| Spdef | A_55_P2007249 | NM_013891 | 9.80 | -1.21 | SAM pointed domain containing ets transcription factor (Spdef) |
| Spon2 | A_52_P381484 | NM_133903 | 9.29 | -1.56 | spondin 2. extracellular matrix protein (Spon2) |
| Sstr3 | A_55_P2106335 | NM_009218 | 7.82 | -1.18 | somatostatin receptor 3 (Sstr3) |
| St3gal1 | A_51_P301804 | NM_009177 | 11.43 | 1.01 | ST3 beta-galactoside alpha-2.3-sialyltransferase 1 (St3gal1) |
| St6galnac1 | A_55_P2118609 | NM_011371 | 12.53 | 1.39 | ST6 (alpha-N-acetyl-neuraminyl-2.3-beta-galactosyl-1.3)-N-acetylgalactosaminide alpha-2.6-sialyltransferase 1 (St6galnac1) |
| Sulf2 | A_52_P193925 | NM_028072 | 12.42 | 1.19 | sulfatase 2 (Sulf2). transcript variant 2 |
| Susd4 | A_55_P1954393 | NM_144796 | 11.41 | 2.69 | sushi domain containing 4 (Susd4) |
| Svop | A_51_P282594 | NM_026805 | 7.88 | 1.62 | SV2 related protein (Svop) |
| Sytl1 | A_55_P2143693 | NM_031393 | 11.81 | 1.46 | synaptotagmin-like 1 (Sytl1) |
| Tacr1 | A_52_P149545 | NM_009313 | 7.39 | -1.21 | tachykinin receptor 1 (Tacr1) |
| Tbx22 | A_55_P2089565 | NM_181319 | 10.46 | 1.81 | T-box 22 (Tbx22). transcript variant 2 |
| Tdgf1 | A_55_P1968028 | NM_011562 | 11.07 | 1.51 | teratocarcinoma-derived growth factor 1 (Tdgf1) |
| Tdrd3 | A_55_P1983036 | NM_172605 | 11.31 | 1.16 | tudor domain containing 3 (Tdrd3). transcript variant 1 |
| Tekt1 | A_55_P2132024 | NM_011569 | 11.61 | -1.71 | tektin 1 (Tekt1) |
| Tepp | A_51_P450682 | NM_199455 | 9.04 | -1.24 | testis. prostate and placenta expressed (Tepp). transcript variant 1 |
| Tgm1 | A_52_P627816 | NM_019984 | 9.37 | 1.73 | transglutaminase 1. K polypeptide (Tgm1). transcript variant 2 |
| Them5 | A_51_P267441 | NM_025416 | 7.41 | 1.52 | thioesterase superfamily member 5 (Them5) |
| Thyn1 | A_51_P323620 | NM_144543 | 13.94 | 1.73 | thymocyte nuclear protein 1 (Thyn1) |
| Tinagl1 | A_52_P418489 | NM_023476 | 12.28 | 1.16 | tubulointerstitial nephritis antigen-like 1 (Tinagl1). transcript variant 1 |
| Tk1 | A_55_P2056496 | NM_009387 | 9.36 | -1.30 | thymidine kinase 1 (Tk1) |
| Tlr2 | A_51_P452629 | NM_011905 | 12.15 | -1.24 | toll-like receptor 2 (Tlr2) |
| Tmem132a | A_55_P2113081 | NM_133804 | 13.61 | 1.36 | transmembrane protein 132A (Tmem132a) |
| Tmem171 | A_52_P799815 | NM_001025606 | 10.22 | -1.66 | transmembrane protein 171 (Tmem171) |
| Tmem213 | A_51_P401501 | NM_029921 | 9.16 | -1.08 | transmembrane protein 213 (Tmem213) |
| Tmprss13 | A_55_P2177998 | NM_001013373 | 10.46 | 1.27 | transmembrane protease. serine 13 (Tmprss13) |
| Tnfrsf10b | A_55_P2027836 | NM_020275 | 10.96 | 1.96 | tumor necrosis factor receptor superfamily. member 10b (Tnfrsf10b) |
| Tnfrsf12a | A_51_P131408 | NM_013749 | 13.08 | 1.17 | tumor necrosis factor receptor superfamily. member 12a (Tnfrsf12a). transcript variant 1 |
| Tnn | A_55_P2005549 | NM_177839 | 8.59 | 1.58 | tenascin N (Tnn) |
| Tob2 | A_52_P70854 | NM_020507 | 12.04 | 1.01 | transducer of ERBB2. 2 (Tob2) |
| Tomm20l | A_51_P518156 | NM_029227 | 7.34 | 2.26 | translocase of outer mitochondrial membrane 20 homolog (yeast)-like (Tomm20l). nuclear gene encoding mitochondrial protein |
| Trib1 | A_52_P573552 | NM_144549 | 12.81 | 1.20 | tribbles homolog 1 (Drosophila) (Trib1) |
| Trim11 | A_55_P1962359 | NM_053168 | 13.14 | 1.38 | tripartite motif-containing 11 (Trim11) |
| Trp53inp1 | A_55_P1973906 | NM_021897 | 9.42 | 2.24 | transformation related protein 53 inducible nuclear protein 1 (Trp53inp1). transcript variant 1 |
| Tsc22d3 | A_55_P1989061 | NM_001077364 | 14.10 | 1.29 | TSC22 domain family. member 3 (Tsc22d3). transcript variant 1 |
| Tspan1 | A_51_P333923 | NM_133681 | 14.65 | -1.65 | tetraspanin 1 (Tspan1) |
| Tspan33 | A_55_P2086664 | NM_146173 | 12.08 | -1.52 | tetraspanin 33 (Tspan33) |
| Tspan4 | A_55_P2007713 | NM_053082 | 11.28 | 1.02 | tetraspanin 4 (Tspan4). transcript variant 1 |
| Vopp1 | A_55_P1970120 | NM_146168 | 12.70 | 1.11 | vesicular. overexpressed in cancer. prosurvival protein 1 (Vopp1) |
| Vsig8 | A_55_P2124233 | NM_177723 | 9.76 | 1.11 | V-set and immunoglobulin domain containing 8 (Vsig8). transcript variant 1 |
| Wdfy4 | A_52_P199614 | NM_001146022 | 9.34 | -2.00 | WD repeat and FYVE domain containing 4 (Wdfy4) |
| Wee1 | A_66_P136186 | NM_009516 | 9.38 | 1.17 | WEE 1 homolog 1 (S. pombe) (Wee1) |
| Wfikkn2 | A_52_P625215 | NM_181819 | 8.54 | -1.55 | WAP. follistatin/kazal. immunoglobulin. kunitz and netrin domain containing 2 (Wfikkn2) |
| Wisp1 | A_51_P220343 | NM_018865 | 11.35 | -2.08 | WNT1 inducible signaling pathway protein 1 (Wisp1) |
| Wnt7b | A_55_P2274592 | NM_001163634 | 7.61 | 1.00 | wingless-related MMTV integration site 7B (Wnt7b). transcript variant 2 |
| Zbtb16 | A_55_P2024155 | NM_001033324 | 13.03 | 1.73 | zinc finger and BTB domain containing 16 (Zbtb16) |
| Zfp365 | A_55_P2039320 | NM_178679 | 10.50 | 3.07 | zinc finger protein 365 (Zfp365) |
| Zfp688 | A_55_P1971759 | NM_026999 | 11.76 | 1.01 | zinc finger protein 688 (Zfp688) |
| Zmat3 | A_51_P415220 | NM_009517 | 11.48 | 1.82 | zinc finger matrin type 3 (Zmat3) |
