# Supplementary material for: 99mTcO4 −-, Auger-Mediated Thyroid Stunning: Dosimetric Requirements and Associated Molecular Events
Source: PLoS One. 2014 Mar 24;9(3):e92729. doi: 10.1371/journal.pone.0092729 (PMC3963936; doi:10.1371/journal.pone.0092729)
Supplement: Table S2 — Ingenuity Pathway Analysis of microarray data to highlight selectively affected pathways in 99mTcO4−-exposed thyroids. Affected canonical pathways ordered by p-value are presented. The probability of obtaining the number of genes in a certain pathway in the list of differentially expressed genes was compared with the representation of the same pathway among all the genes on the microarray; –log10 of the Fisher's exact probability is indicated. (DOCX) [file pone.0092729.s002.docx]

**Supplemental Table 2: Ingenuity Pathway Analysis of microarray data to highlight selectively affected pathways in ^99m^TcO_4_^−^-exposed thyroids.**

| **Canonical Pathways** | **-log(p value)** | **Molecules** |
| --- | --- | --- |
| p53 Signaling | 9.84 | CCNG1, BCL2L1, MDM4, TP53INP1, BBC3, GADD45G, CDKN1A, APAF1, PERP, RPRM, MDM2, BAX, PIDD, TNFRSF10A, FAS |
| Autoimmune Thyroid Disease Signaling | 4.53 | HLA-DRA, HLA-DQA1, HLA-DRB1, CD86, HLA-DQB1, FAS |
| Graft-versus-Host Disease Signaling | 4.22 | HLA-DRA, HLA-DQA1, HLA-DRB1, CD86, HLA-DQB1, FAS |
| B Cell Development | 4.08 | HLA-DRA, HLA-DQA1, HLA-DRB1, CD86, HLA-DQB1 |
| Cell Cycle: G2/M DNA Damage Checkpoint Regulation | 3.84 | MDM4, WEE1, CDKN1A, CKS1B, MDM2, RPRM |
| Allograft Rejection Signaling | 3.72 | HLA-DRA, HLA-DQA1, HLA-DRB1, CD86, HLA-DQB1, FAS |
| Nur77 Signaling in T Lymphocytes | 3.61 | HLA-DRA, HLA-DQA1, APAF1, HLA-DRB1, CD86, HLA-DQB1 |
| Cytotoxic T Lymphocyte-mediated Apoptosis of Target Cells | 3.46 | HLA-DRA, HLA-DQA1, APAF1, HLA-DRB1, HLA-DQB1, FAS |
| Altered T Cell and B Cell Signaling in Rheumatoid Arthritis | 3.18 | TLR2, HLA-DRA, HLA-DQA1, HLA-DRB1, CD86, HLA-DQB1, FAS |
| Type I Diabetes Mellitus Signaling | 3.15 | SOCS1, HLA-DRA, HLA-DQA1, APAF1, HLA-DRB1, CD86, HLA-DQB1, FAS |
| IL-4 Signaling | 2.67 | SOCS1, HLA-DRA, HLA-DQA1, HLA-DRB1, HLA-DQB1, JAK3 |
| Dendritic Cell Maturation | 2.63 | TLR2, COL5A3, HLA-DRA, HLA-DQA1, HLA-DRB1, CD86, IRF8, HLA-DQB1, PLCD4 |
| OX40 Signaling Pathway | 2.42 | BCL2L1, HLA-DRA, HLA-DQA1, HLA-DRB1, HLA-DQB1 |
| Induction of Apoptosis | 2.28 | BCL2L1, BBC3, APAF1, BAX, FAS |
| CTLA4 Signaling in Cytotoxic T Lymphocytes | 2.26 | HLA-DRA, HLA-DQA1, HLA-DRB1, CD86, HLA-DQB1, PTPN22 |
| Docosahexaenoic Acid (DHA) Signaling | 2.22 | BCL2L1, APAF1, BAX, BCL2A1 |
| Apoptosis Signaling | 2.17 | BCL2L1, CAPN6, APAF1, BAX, BCL2A1, FAS |
| T Helper Cell Differentiation | 2.13 | HLA-DRA, HLA-DQA1, HLA-DRB1, CD86, HLA-DQB1 |

|  |
| --- |
