# Supplementary material for: 99mTcO4 −-, Auger-Mediated Thyroid Stunning: Dosimetric Requirements and Associated Molecular Events
Source: PLoS One. 2014 Mar 24;9(3):e92729. doi: 10.1371/journal.pone.0092729 (PMC3963936; doi:10.1371/journal.pone.0092729)
Supplement: Material S1 — Dosimetric calculations. (DOCX) [file pone.0092729.s003.docx]

**Dosimetric report**

1. **Materials and Methods**

Dosimetric assessments were based on the MIRD formalism (*1*) which expresses the absorbed dose calculation as:

where:

  $\bar{D}_{k}$ mean absorbed dose in gray (Gy) to the target k from the radiation emitted by all sources h;

$\tilde{A}_{h}$ cumulated activity in becquerel second (Bq.s) in source h;

$S_{(k\leftarrow h)}$ mean absorbed dose in gray per becquerel-second (Gy.Bq^-1^.s^-1^) to the target k per unit of cumulated activity in source h (or S-value)

$\tilde{A}_{h}$ was calculated from pharmacokinetics data detailed in the paper.

Self S-values $S_{(h\leftarrow h)}$ were calculated by Monte Carlo simulation for each radionuclide and were based on a representative mouse model.

- 1. **Pharmacokinetic data**

Activities located in the thyroid and used for calculation were taken from the standard measurement method, consisting of measuring the activity in the whole region of interest, with no correction for nuclear decay. Cumulated activity was calculated for each mouse using a linear fitting model for the uptake phase, and a mono-exponential fitting model for the washout, both implemented in Root software (<http://root.cern.ch>).

- 1. **Geometric mouse model**

This study was based on the realistic digital mouse (Moby) whole-body phantom (version 2), representing a 16-week-old male C57BL/6 mouse (*2*). This model is based on non uniform rational B-spline (NURBS) mathematical models and allows flexible manipulation of animal organs and body by defining a set of control points on each surface. The model is provided to the user as an interactive program that allows one or more selected organs to be scaled. Therefore, we generated a 22 g mouse model as a three-dimensional rectangular matrix of cubic voxels (200x200x200 µm^3^). The final 3D-image dataset was composed of 256x550x256 voxels and saved in raw format (16-bit; unsigned integer; little-endian; 72 MB). Thyroid mass was reduced to 5.4 mg by applying an erode mask implemented on ImageJ software. Other parameters used to define the model are listed in Table **1**.

| **software model** | Moby |
| --- | --- |
| **software version** | v2 |
| **total body mass (g)** | 22 |
| **spatial sampling (µm^3^)** | 200x200x200 |
| **media** | soft tissue, lungs, bones, air |
| **density and material composition** | Cristy & Eckerman (*3*) |

Table 1: description of parameters used to define the geometric model

- 1. **Dosimetric calculation**
     1. **S-values**

S-values were calculated using Monte Carlo modelling of radiation transport and energy deposition in the voxel-based mouse model. Up to 10^6^ particles were simulated using the recent version of GATE (6.1) and based on GEANT4 toolkit (version 9.04 patch01) well-established codes for radiation transport (*4-6*).

The voxel-based mouse was implemented with the *CompressedMatrix* option, which was the most suited function available for dosimetric purposes in that version, and regions of interest were defined using the *range* option. *Physics List Standard Option 3* was used to define physics processes. Production cuts for electrons and gamma were set to be equivalent to 1 keV in soft tissues. The deposited energy was scored at the voxel level of the phantom with the DoseActor *doseDistributionEdep*. Statistical uncertainties were calculated using the associated *UncertaintyEdep* option.

The dosel grid (scoring matrix volume) (*7*) was of the same size as the phantom matrix and the output energy distribution was generated as a three-dimensional voxel-based map. Ionization steps for electrons and positrons were set to 1/20 of the dosel size. GATE was run with Mersenne Twister (*8*) random number generator.

In order to speed up calculations, a high performance cluster (20 Xeon Westmere 12-core with 16 Gb RAM each and a 16Tb archive system) with 480 virtual cores was used to perform fast and accurate Monte Carlo simulations. Parallel calculation was supported by the Application Programming Interface (API) Xgrid developed by Apple and post processing was performed with ImageJ (*9*).

Thyroid self S-values were calculated for ^99m^Tc. All detailed photon and electron emissions were based on the “MIRD radionuclide data and decay schemes” (*10*). Mean emitted energies and numbers of particles per nuclear transition are listed in Table **2** for each emission type.

Cubic spline interpolation was applied to all continuous energy spectra used in simulations. Sources were assumed to be distributed homogeneously within the thyroid and statistical uncertainties on self S-values were kept below 1%.

| **photons** | **mean energy per nt.**  **(MeV.Bq^-1^.s^-1^)** | 1.266E^-01^ |
| --- | --- | --- |
|  | **number of particles per nt. (Bq.s)** | 6.467E^+00^ |
| **electrons + ß^-^** | **mean energy per nt. (MeV.Bq^-1^.s^-1^)** | 1.619E^-02^ |
|  | **number of particles per nt. (Bq.s)** | 5.517E^+00^ |
| **total** | **mean energy per nt.**  **(MeV.Bq^-1^.s^-1^)** | 1.428E^-01^ |
|  | **number of particles per nt. (Bq.s)** | 1.198E^+01^ |

Table 2: Nuclear data for ^99m^Tc taken from ref (*10*) – nt.: nuclear transition.

- - 1. **Absorbed fraction**

We also calculated the absorbed fraction $\varphi=\frac{E}{E_{0}}$, i.e. the ratio of energy $E$ deposited in the thyroid by the theoretical energy $E_{0}$ emitted in the thyroid.

1. **Results**

Table **3** contains the absorbed fraction of energy that is deposited in the thyroid of the representative mouse model as well as the thyroid self S-value for ^99m^Tc.

| **radionuclide** | **^99m^Tc** |
| --- | --- |
| **absorbed fraction** | 0.109 |
| **thyroid self S-value (Gy.Bq^-1^.s^-1^)** | 4.55E^-10^ |

Table 3: thyroid absorbed fraction and self S-value for ^99m^Tc.

Table **4** contains the mean absorbed dose in the thyroid for both control and special diet groups within the first 24 hours after injection of ^99m^Tc.

| **^99m^Tc** | |
| --- | --- |
| **experimental group** | **mean absorbed dose (Gy)** |
| special diet | 47.6 [2.9] |
| control | 23.6 [6.6] |

Table 4: thyroid self-absorbed dose calculated for each mouse within the first 24 hours for both ^123^I and ^99m^Tc – [standard deviation]References

**1.** Loevinger R, Budinger TF, Watson EE. MIRD primer for absorbed dose calculations, Revised. New York: The Society of Nuclear Medicine; 1991.

**2.** Segars WP, Tsui BM, Frey EC, Johnson GA, Berr SS. Development of a 4-D digital mouse phantom for molecular imaging research. *Mol Imaging Biol.* May-Jun 2004;6(3):149-159.

**3.** Cristy M, Eckerman KF. Specific absorbed fractions of energy at various ages for internal photon sources. *Oak Ridge, TN: Oak Ridge National Lab.* 1987(ORNL/TM-8381 ).

**4.** Jan S, Santin G, Strul D, et al. GATE: a simulation toolkit for PET and SPECT. *Phys Med Biol.* Oct 7 2004;49(19):4543-4561.

**5.** Jan S, Benoit D, Becheva E, et al. GATE V6: a major enhancement of the GATE simulation platform enabling modelling of CT and radiotherapy. *Phys Med Biol.* Feb 21 2011;56(4):881-901.

**6.** Agostinelli S, Allison J, Amako K. GEANT4-a simulation toolkit. *Nucl Inst Meth Phys Res.* 2003;A506:250-303.

**7.** Sarrut D, Guigues L. Region-oriented CT image representation for reducing computing time of Monte Carlo simulations. *Med Phys.* Apr 2008;35(4):1452-1463.

**8.** Matsumoto M, Nishimura T. Mersenne twister: a 623-dimensionally equidistributed uniform pseudo-random number generator. *ACM Transactions on Modeling and Computer Simulation.* 1998;8(1).

**9.** Rasband WS. ImageJ : US National Institutes of Health, Bethesda, Maryland. *MD: US National Institutes of Health See* [*http://rsbinfonihgov/ij*](http://rsbinfonihgov/ij)*.* 1997.

**10.** Eckerman KF, Endo A. *MIRD:Radionuclide Data and Decay Schemes* Society for Nuclear Medicine; 2008.
